# Ablation of α_2_δ-1 inhibits cell-surface trafficking of endogenous N-type calcium channels in the pain pathway in vivo

**DOI:** 10.1073/pnas.1811212115

**Published:** 2018-11-28

**Authors:** Manuela Nieto-Rostro, Krishma Ramgoolam, Wendy S. Pratt, Akos Kulik, Annette C. Dolphin

**Affiliations:** ^a^Department of Neuroscience, Physiology and Pharmacology, University College London, WC1E 6BT London, United Kingdom;; ^b^Institute of Physiology II, Faculty of Medicine, University of Freiburg, 79104 Freiburg, Germany;; ^c^BIOSS Centre for Biological Signalling Studies, University of Freiburg, 79104 Freiburg, Germany

**Keywords:** calcium channel, primary afferent, auxiliary subunit, N-type, trafficking

## Abstract

The auxiliary α_2_δ calcium channel subunits play key roles in voltage-gated calcium channel function. Independent of this, α_2_δ-1 has also been suggested to be important for synaptogenesis. Using an epitope-tagged knockin mouse strategy, we examined the effect of α_2_δ-1 on Ca_V_2.2 localization in the pain pathway in vivo, where Ca_V_2.2 is important for nociceptive transmission and α_2_δ-1 plays a critical role in neuropathic pain. We find Ca_V_2.2 is preferentially expressed on the plasma membrane of calcitonin gene-related peptide-positive small nociceptors. This is paralleled by strong presynaptic expression of Ca_V_2.2 in the superficial spinal cord dorsal horn. EM-immunogold localization shows Ca_V_2.2 predominantly in active zones of glomerular primary afferent terminals. Genetic ablation of α_2_δ-1 abolishes Ca_V_2.2 cell-surface expression in dorsal root ganglion neurons and dramatically reduces dorsal horn expression. There was no effect of α_2_δ-1 knockout on other dorsal horn pre- and postsynaptic markers, indicating the primary afferent pathways are not otherwise affected by α_2_δ-1 ablation.

## Significance

Neuronal N-type (Ca_V_2.2) voltage-gated calcium channels are important at the first synapse in the pain pathway. In this study, we have characterized a knockin mouse containing Ca_V_2.2 with an extracellular HA tag to determine the localization of Ca_V_2.2 in primary afferent pain pathways. These endogenous channels have been visualized at the plasma membrane and rigorously quantified in vivo. We examined the effect of ablation of the calcium channel auxiliary subunit α_2_δ-1 (the target of gabapentinoids) on Ca_V_2.2 distribution. We found preferential cell-surface localization of Ca_V_2.2 in DRG nociceptor neuron cell bodies was lost, accompanied by a dramatic reduction at dorsal horn terminals, but no effect on distribution of other spinal cord synaptic markers.

The neuronal N-type voltage-gated calcium channel was first identified in primary afferent dorsal root ganglion (DRG) neurons (1, 2). Toxins from the *Conus* marine snails, ω-conotoxin GVIA and ω-conotoxin MVIIC, are highly selective blockers of N-type channels (3, 4) and have been instrumental in dissecting their function (5, 6). A key role for N-type calcium channels was identified in primary afferent neurotransmission in the dorsal horn of the spinal cord, and these toxins were therefore pursued as a therapeutic target in the alleviation of chronic pain (7, 8). Indeed, the peptide ziconotide (synthetic ω-conotoxin MVIIA) is licensed for intrathecal use in intractable pain conditions (9, 10).

Despite the functional importance of N-type channels in the pain pathway, a major hindrance to the study of their distribution and trafficking, in this system and elsewhere, has been the paucity of antibodies recognizing this channel. Although previous studies have used anti-peptide antibodies to intracellular Ca_V_2.2 epitopes (for example, refs. 11 and 12), these have not shown plasma membrane localization of the endogenous channel in neurons and have not been rigorously examined against knockout tissue. For this reason, we developed a Ca_V_2.2 construct with an exofacial epitope tag to detect its cell-surface expression and trafficking (13). This channel is observed on the plasma membrane, when expressed in DRGs and other neurons (13–15). We took advantage of our finding that the presence of the epitope tag did not affect function (13) to generate a knockin (KI) mouse line containing the hemagglutinin (HA) tag in the same position in the *Cacna1b* gene. This has allowed us to examine the distribution of native Ca_V_2.2 protein in the intact nervous system.

N-type calcium channels are made up of the Ca_V_2.2 pore-forming α1-subunit, which associates with auxiliary α_2_δ- and β-subunits (16). Many studies have indicated that α_2_δ-subunits are important for the correct trafficking and physiological function of the channels (for a review, see ref. 17). A significant role for α_2_δ-1 in chronic neuropathic pain, which results from damage to peripheral sensory nerves, was identified as a result of two advances. First, it was shown that α_2_δ-1 mRNA and protein are strongly up-regulated in somatosensory neurons following nerve damage (18–20). Second, α_2_δ-1 was identified as the therapeutic target for the drugs gabapentin and pregabalin, which are used in neuropathic pain such as postherpetic neuralgia (21, 22). Furthermore, α_2_δ-1 overexpression in mice resulted in a chronic pain-like phenotype (23), whereas knockout of α_2_δ-1 caused a marked delay in the development of neuropathic mechanical hypersensitivity (24). However, it has not yet been possible to examine the effect of α_2_δ-1 on the trafficking of the relevant N-type channels in vivo.

Here we elucidate the cellular and subcellular localization of native Ca_V_2.2 in neurons of the peripheral somatosensory nervous system. We reveal a dramatic effect of α_2_δ-1 ablation on Ca_V_2.2 distribution, particularly in a key subset of nociceptive sensory neurons. In contrast to an early study of the subunit composition of N-type channels (16), which showed an ∼1:1 stoichiometry with α_2_δ-1, a more recent study suggested that α_2_δ-subunits were only associated with less than 10% of digitonin-solubilized Ca_V_2 channels (25), although it cannot be ruled out that they became dissociated during solubilization. However, the present study reinforces the essential nature of the auxiliary α_2_δ-1 protein for cell-surface expression of endogenous Ca_V_2.2, both in DRG neuronal cell bodies and in their presynaptic terminals. No effect of α_2_δ-1 loss was observed on other pre- and postsynaptic markers in the dorsal horn, despite a previous study implicating postsynaptic α_2_δ-1 in thrombospondin-mediated synaptogenesis (26). Our results therefore show that loss of synaptic Ca_V_2.2 as a result of α_2_δ-1 ablation is due to a reduction of Ca_V_2.2 trafficking to synapses, rather than synapse loss.

## Results

### Generation of Ca_V_2.2_HA Knockin Mice.

**Fig. 1.**
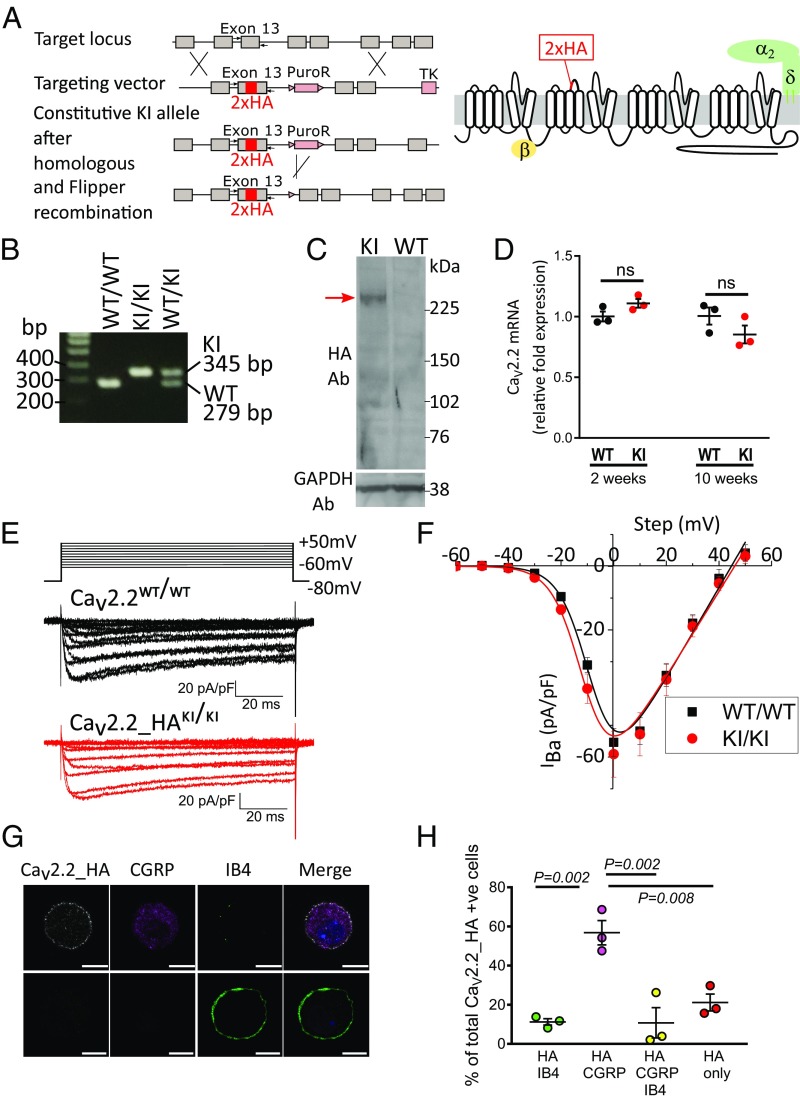
Characterization of Ca_V_2.2_HA knockin mice. (*A*, *Left*) Strategy for generation of Ca_V_2.2_HA^KI/KI^ mice. (*A*, *Right*) Diagram of Ca_V_2.2 showing the position of the HA tag. (*B*) Genotyping showing PCR product size for Ca_V_2.2^WT/WT^, Ca_V_2.2_HA^KI/KI^, and Ca_V_2.2_HA^KI/WT^ mice, using the primers shown in *A*. (*C*) Immunoblot of spinal cord synaptosomes from Ca_V_2.2_HA^KI/KI^ mice (*Left*) and Ca_V_2.2^WT/WT^ mice (*Right*), confirming expression of Ca_V_2.2_HA at the expected size (red arrow). GAPDH (*Lower*) is the loading control. Representative of three independent experiments from different mice. The molecular mass of Ca_V_2.2_HA is 261.0 ± 1.2 kDa. (*D*) qPCR for Ca_V_2.2 mRNA in brains from Ca_V_2.2_HA^KI/KI^ (red circles), compared with Ca_V_2.2^WT/WT^ (black circles), at 2 and 10 wk postnatally (*n* = 3 mice per condition, each assayed in triplicate; one outlier triplicate value was omitted). Mean ± SEM is also shown. ns, not significant, paired *t* test. (*E*) Representative calcium channel currents recorded from Ca_V_2.2^WT/WT^ (black traces) and Ca_V_2.2_HA^KI/KI^ (red traces) DRG neurons in culture (1 d in vitro). Currents were recorded at 10-mV intervals from −60 to + 50 mV. Capacitance transients have been cropped. (*F*) Current–voltage (IV) relationships (mean ± SEM) for *I*_Ba_ from Ca_V_2.2_HA^KI/KI^ (red circles; *n* = 39 cells from four mice) and Ca_V_2.2^WT/WT^ (black squares; *n* = 37 cells from four mice) DRG neurons. Data were fit with a modified Boltzmann relationship (*SI Appendix*, *Methods*). For Ca_V_2.2_HA^KI/KI^ and Ca_V_2.2_HA^WT/WT^, the parameters for the illustrated fits are *V*_50,act_ −10.69 and −8.00 mV; *G*_max_ 1.34 and 1.42 nS.pF^−1^; and *V*_rev_ +45.9 and +44.7 mV, respectively. For the individual data for Ca_V_2.2_HA^KI/KI^ (*n* = 39) and Ca_V_2.2_HA^WT/WT^ (*n* = 37), *V*_50,act_ was −9.87 ± 0.62 and −8.30 ± 0.48 mV; *G*_max_ was 1.31 ± 0.15 and 1.43 ± 0.09 nS.pF^−1^; and *V*_rev_ was +44.0 ± 1.5 and +44.3 ± 1.5 mV, respectively. None of the parameters show any statistical difference (Student’s *t* test). (*G*) Images of cultured DRG neurons from Ca_V_2.2_HA^KI/KI^ mice showing (*Left* to *Right*) Ca_V_2.2_HA staining before permeabilization, CGRP staining following permeabilization, IB4-FITC, and merged for two representative CGRP-positive (*Top*) and IB4-positive (*Bottom*) cells. (Scale bars: 10 μm.) (*H*) Quantification of the percentage of cells with cell-surface Ca_V_2.2_HA that were also positive for IB4 (green circles), CGRP (lilac circles), CGRP and IB4 (yellow circles), or neither marker (red circles). Individual data points represent the mean data from three separate experiments and a total of 206 DRG neurons. Mean ± SEM of the three experiments is superimposed. Statistical significances compared with HA + CGRP-containing DRG neurons are shown (one-way ANOVA and Sidak’s multiple-comparisons test).

Mice containing a double-HA tag in constitutive exon 13 of the *Cacna1b* gene were generated in a C57BL/6 background, as described in *Methods*, such that every endogenous Ca_V_2.2 contained the double-HA tag in the position previously ascertained not to affect channel function (13) (Fig. 1*A*). The presence of the HA tag was confirmed by PCR (Fig. 1*B*). We confirmed that the HA-tagged Ca_V_2.2 protein is expressed in synaptosomes, since a 261-kDa band (the expected molecular mass of Ca_V_2.2_HA) is recognized by anti-HA antibodies in Western blots of spinal cord tissue from Ca_V_2.2_HA^KI/KI^, but not Ca_V_2.2^WT/WT^, mice (Fig. 1*C*).

### Ca_V_2.2 mRNA Levels and Calcium Currents Are Unaltered in Ca_V_2.2_HA^KI/KI^ Compared with Ca_V_2.2^WT/WT^ Mice.

We next confirmed that the expression of Ca_V_2.2 did not differ between Ca_V_2.2_HA^KI/KI^ and Ca_V_2.2^WT/WT^ mice. The analyzed expression profiles at 2 and 10 wk postnatally showed that Ca_V_2.2 mRNA levels were not altered in the Ca_V_2.2_HA^KI/KI^ compared with Ca_V_2.2^WT/WT^ mouse brains (Fig. 1*D*).

The properties of calcium channel currents in cultured DRG neurons from 10- to 12-wk-old Ca_V_2.2_HA^KI/KI^ mice were not altered compared with those from Ca_V_2.2^WT/WT^ mice, both in terms of current density and voltage-dependent properties (Fig. 1 *E* and *F*). We then examined whether Ca_V_2.2_HA was detectable on the cell surface of cultured DRG neurons from Ca_V_2.2_HA^KI/KI^ mice (Fig. 1*G*). We found Ca_V_2.2_HA to be present on the cell surface particularly of calcitonin gene-related peptide (CGRP)-positive peptidergic nociceptors, to a much greater extent than on isolectin-B4 (IB4)–positive nonpeptidergic nociceptors (56.8%, compared with 11.3%; Fig. 1 *G* and *H*). Furthermore, Ca_V_2.2_HA was expressed on only a small proportion of neurofilament 200 (NF200)-positive DRG neurons (77.4% of HA-positive cells were NF200-negative; *SI Appendix*, Fig. S1 *A* and *B*). HA immunostaining was absent from DRG neurons cultured from wild-type mice (*SI Appendix*, Fig. S1*C*).

### Cell-Surface Expression of Ca_V_2.2_HA in DRG Neurons in Vivo.

**Fig. 2.**
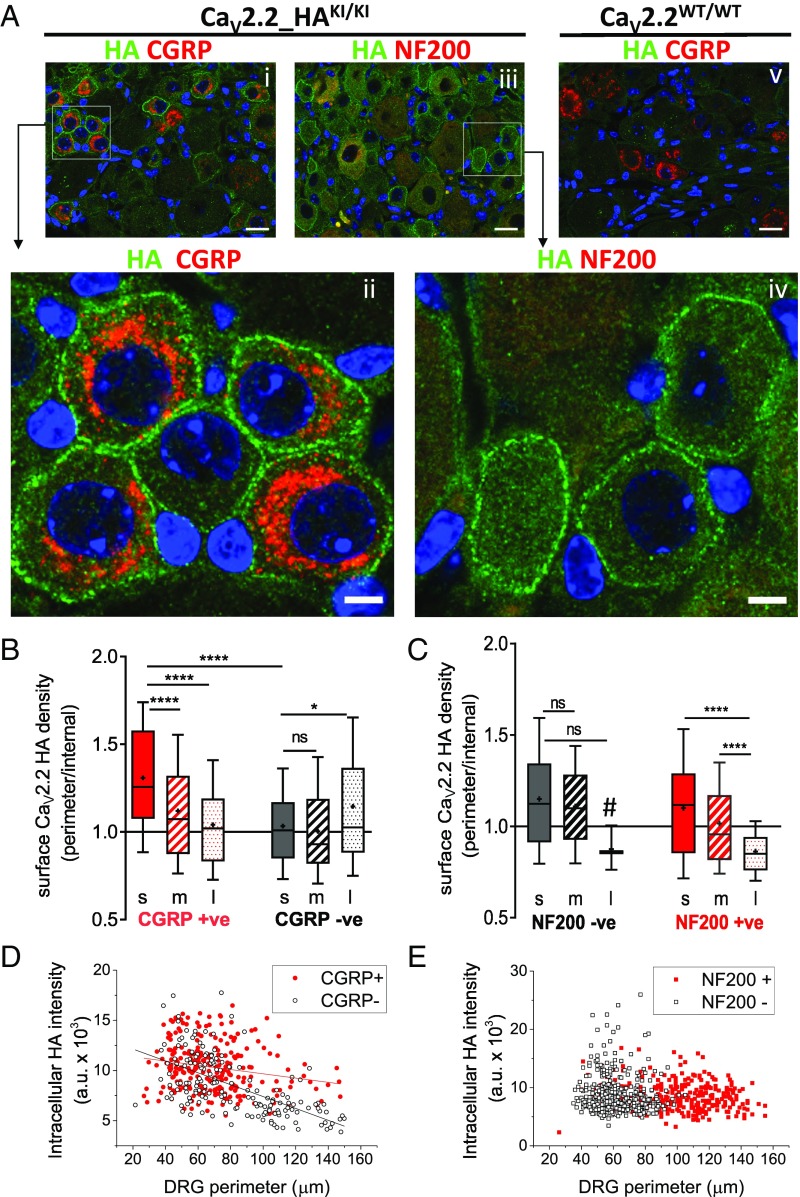
Distribution of Ca_V_2.2_HA in intact dorsal root ganglia. (*A*) Immunostaining for HA (green) in Ca_V_2.2_HA^KI/KI^ (*i*–*iv*) and WT DRG neurons (*v*), costained with CGRP (*i*, *ii*, and *v*) or NF200 (*iii* and *iv*) (red). Nuclei are stained with DAPI (blue). Images *ii* and *iv* are enlargements of the ROIs shown in *i* and *iii*. [Scale bars: 20 (*i*, *iii*, and *v*) and 5 µm (*ii* and *iv*).] Lack of HA staining in Ca_V_2.2^WT/WT^ sections (*v*) was observed in four additional independent experiments. (*B*) Surface Ca_V_2.2_HA intensity measured as a ratio of DRG neuronal perimeter/cytoplasmic staining for small (s; solid bars), medium (m; hatched bars), and large (l; shaded bars) DRG neurons that are either CGRP-positive (red) or CGRP-negative (black/dark gray). *n* = 313, 341, 122, 171, 169, and 164 DRG neurons, respectively, from sections from at least three mice. ****P < 0.0001, *P = 0.0135 (one-way ANOVA and Sidak’s post hoc test of all conditions with correction for multiple comparisons); ns, not significant. (*C*) Surface Ca_V_2.2_HA intensity measured as a ratio of DRG neuronal perimeter/cytoplasmic staining for small, medium, and large DRG neurons that are either NF200-negative (black) or NF200-positive (red). *n* = 213, 201, 3, 36, 97, and 192 DRG neurons, respectively, from sections from at least three mice. #: Note very few large DRG neurons are NF200-negative. ****P < 0.0001 (one-way ANOVA and Sidak’s post hoc test of all conditions with correction for multiple comparisons); ns, not significant. (*D* and *E*) Intracellular Ca_V_2.2_HA staining, quantified with respect to cell size, for CGRP-positive (red circles) and CGRP-negative (open black circles) DRG neurons (*D*), and for NF200-positive (red squares) and NF200-negative (open black squares) DRG neurons (*E*). Data are from a subset of experiments from *B* performed in parallel in which the absolute immunostaining levels are directly comparable. Linear regression analysis for data in *D* (red line, CGRP +ve: slope −21.2, *r*^2^ = 03989, *df* 260, *F* = 10.8, *P* = 0.0012; black line, CGRP −ve: slope −59.2, *r*^2^ = 0.362, *df* 168, *F* = 95.3, *P* < 0.0001).

In agreement with the results from cultured DRG neurons, we found that Ca_V_2.2_HA was clearly present on the cell surface of DRG neuronal somata in sections of ganglia from 10- to 12-wk-old Ca_V_2.2_HA^KI/KI^ mice (Fig. 2 *A*, *i*–*iv*), and absent from Ca_V_2.2^WT/WT^ mice (Fig. 2 *A*, *v*). We costained with markers of DRG neuronal subtypes, including CGRP (Fig. 2 *A*, *i*, *ii*, and *v*) and NF200 (Fig. 2 *A*, *iii* and *iv*). Analysis of the ratio of Ca_V_2.2_HA at the cell perimeter, relative to its cytoplasmic staining, shows that plasma membrane Ca_V_2.2_HA density is highest on the cell surface of small CGRP-positive DRG neurons (Fig. 2*B*). The small cell-surface Ca_V_2.2_HA-positive DRG neurons were mainly NF200-negative (Fig. 2*C*). The absolute level of cytoplasmic staining of Ca_V_2.2_HA was also negatively correlated with the size of DRG neurons (Fig. 2*D*), being higher in small-diameter neurons and in those which are CGRP-positive (Fig. 2*D*) and NF200-negative (Fig. 2*E*).

### Knockout of α_2_δ-1 Abolishes Cell-Surface Expression of Ca_V_2.2_HA on DRG Neurons in Vivo.

**Fig. 3.**
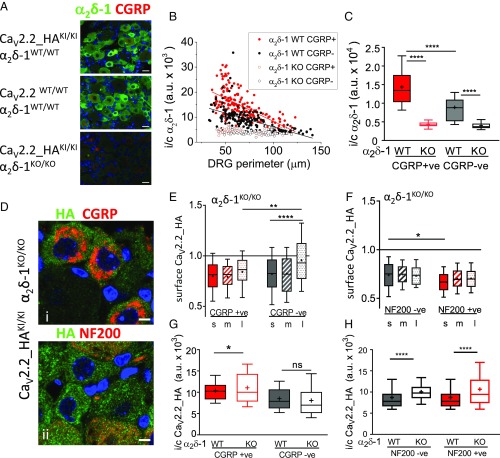
Effect of α_2_δ-1 ablation on distribution of Ca_V_2.2_HA in intact dorsal root ganglia. (*A*) Immunostaining for α_2_δ-1 (green) in Ca_V_2.2_HA^KI/KI^ α_2_δ-1^WT/WT^ (*Top*), Ca_V_2.2^WT/WT^ α_2_δ-1^WT/WT^ (*Middle*), and Ca_V_2.2_HA^KI/KI^ α_2_δ-1^KO/KO^ (*Bottom*) DRG sections, costained with CGRP (red). Nuclei were stained with DAPI (blue). (Scale bars: 20 µm.) (*B*) Intracellular (i/c) α_2_δ-1 density with respect to cell size, for CGRP-positive (red circles) and CGRP-negative (black circles) DRG neurons from Ca_V_2.2_HA^KI/KI^ α_2_δ-1^WT/WT^ (solid circles) and Ca_V_2.2_HA^KI/KI^ α_2_δ-1^KO/KO^ (open circles) mice. Lines are linear fits for both CGRP-positive (red line; *r*^2^ = −0.411; *df* 173, *F* = 120.9, *P* < 0.0001) and CGRP-negative (black line; *r*^2^ = 0.380; *df* 177, *F* = 108.3, *P* < 0.0001) α_2_δ-1^WT/WT^ DRG neurons. (*C*) Intracellular α_2_δ-1 density for CGRP-positive (red bars) and CGRP-negative (black/gray bars) DRG neurons from Ca_V_2.2_HA^KI/KI^ α_2_δ-1^WT/WT^ (solid bars) and Ca_V_2.2_HA^KI/KI^ α_2_δ-1^KO/KO^ (open bars) mice. *n* = 175, 52, 179, and 72 DRG neurons, respectively, from three sections (one mouse per genotype). ****P < 0.0001 (one-way ANOVA and Bonferroni’s post hoc comparison of selected conditions). (*D*) Immunostaining for HA (green) in Ca_V_2.2_HA^KI/KI^ α_2_δ-1^KO/KO^ DRG neurons, costained with CGRP (*i*) or NF200 (*ii*) (red). Nuclei were stained with DAPI (blue). (Scale bars: 20 µm.) (*E*) Surface Ca_V_2.2_HA intensity in α_2_δ-1^KO/KO^ DRG neurons (ratio of perimeter/cytoplasmic staining) for small, medium, and large DRG neurons that are either CGRP-positive (red) or CGRP-negative (black/gray). *n* = 198, 197, 70, 134, 97, and 109 DRG neurons, respectively, from sections from at least three mice. ****P < 0.0001, **P = 0.0022 (one-way ANOVA and Sidak’s post hoc test). (*F*) Surface Ca_V_2.2_HA intensity (ratio of perimeter/cytoplasmic staining) for small, medium, and large DRG neurons that are either NF200-negative (black/gray) or NF200-positive (red). *n* = 204, 136, 5, 37, 94, and 168 DRG neurons, respectively, from sections from at least three mice. *P = 0.0482 (one-way ANOVA and Sidak’s post hoc test). (*G*) Intracellular Ca_V_2.2_HA intensity for CGRP-positive (red) and CGRP-negative (black/gray) α_2_δ-1^WT/WT^ (solid bars) and α_2_δ-1^KO/KO^ (open bars) DRG neurons. *n* = 262, 232, 170, and 144 DRG neurons, respectively. Data are from a subset of experiments performed in parallel in which the absolute immunostaining levels are directly comparable. *P = 0.0308 (one-way ANOVA and Sidak’s post hoc test), ns, not significant. (*H*) Intracellular Ca_V_2.2_HA intensity for NF200-negative (black/gray) and NF200-positive (red) α_2_δ-1^WT/WT^ (solid bars) and α_2_δ-1^KO/KO^ (open bars) DRG neurons. *n* = 417, 345, 325, and 299 DRG neurons, respectively. ****P < 0.0001 (one-way ANOVA and Sidak’s post hoc test).

To determine the importance of α_2_δ-1 in the cell-surface expression of Ca_V_2.2_HA, we crossed Ca_V_2.2_HA^KI/KI^ mice with α_2_δ-1^KO/WT^ mice, and compared Ca_V_2.2_HA^KI/KI^ x α_2_δ-1^KO/KO^ with their Ca_V_2.2_HA^KI/KI^ x α_2_δ-1^WT/WT^ littermates. We first confirmed that DRG neurons from Ca_V_2.2_HA^KI/KI^ x α_2_δ-1^WT/WT^ mice have similar levels of α_2_δ-1 to Ca_V_2.2^WT/WT^ x α_2_δ-1^WT/WT^ mice (Fig. 3*A*; quantified in *SI Appendix*, Fig. S2*A*). We found the level of α_2_δ-1 to be highest in CGRP-positive small DRG neurons (Fig. 3 *A*–*C* and *SI Appendix*, Fig. S2*A*). As expected, Ca_V_2.2_HA^KI/KI^ x α_2_δ-1^KO/KO^ DRG neurons show no staining for α_2_δ-1 above background (Fig. 3 *A*–*C*).

The effect of genetic ablation of α_2_δ-1 on Ca_V_2.2_HA cell-surface expression was in general very marked (Fig. 3 *D*–*F*). We found that Ca_V_2.2_HA was not concentrated on the cell surface in α_2_δ-1^KO/KO^ DRG neurons (Fig. 3*D*), and this was true across all subtypes of DRG neuron examined (Fig. 3 *E* and *F*). Furthermore, there was an increase in mean intracellular Ca_V_2.2_HA intensity in DRG neurons from α_2_δ-1^KO/KO^ compared with α_2_δ-1^WT/WT^ mice, which was found in CGRP-positive DRG neurons (6.9% increase; Fig. 3*G*), and in both NF200-negative and NF200-positive DRG neurons (15.3 and 24.6% increase, respectively; Fig. 3*H*). The elevated intracellular Ca_V_2.2_HA intensity in α_2_δ-1^KO/KO^ DRG neurons was also inversely correlated with cell size (*SI Appendix*, Fig. S2*B*).

### Ca_V_2.2_HA Is Localized in the Dorsal Horn of the Spinal Cord.

**Fig. 4.**
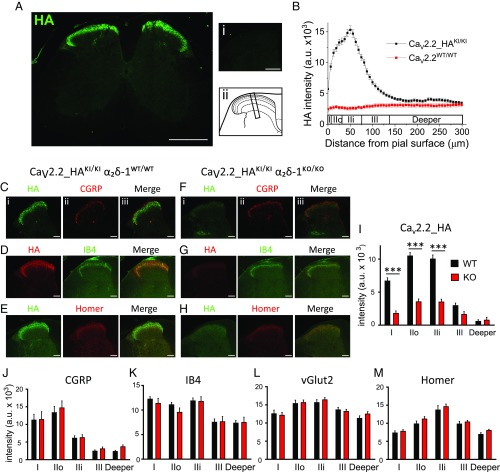
Effect of α_2_δ-1 ablation on distribution of Ca_V_2.2_HA and other synaptic markers in the dorsal horn. (*A*) HA immunostaining (green) in a complete spinal cord section from a Ca_V_2.2_HA^KI/KI^ mouse. (*A*, *Right*) (*i*) Lack of staining in the WT spinal cord, and (*ii*) dorsal horn laminae and ROI perpendicular to the pial surface. [Scale bars: 500 and 200 µm (*i*).] (*B*) Ca_V_2.2_HA intensity (mean ± SEM) in ROIs from the pial surface to 300 µm in the dorsal horn from Ca_V_2.2_HA^KI/KI^ (black squares; *n* = 72 ROIs) and WT (red squares; *n* = 36 ROIs) mice (six ROIs for three experiments for four Ca_V_2.2_HA^KI/KI^ and two Ca_V_2.2^WT/WT^ mice). (*C*–*H*) Dorsal horn HA immunostaining (*Left*, *i*), costained with CGRP (*C* and *F*), IB4 (*D* and *G*), or Homer (*E* and *H*) (*Middle*, *ii*), for Ca_V_2.2_HA^KI/KI^ α_2_δ-1^WT/WT^ (*C*–*E*) and Ca_V_2.2_HA^KI/KI^ α_2_δ-1^KO/KO^ (*F*–*H*) mice. Merged images (*Right*, *iii*). (Scale bars: 100 µm.) (*I*–*M*) Immunostaining in the dorsal horn from α_2_δ-1^WT/WT^ (black bars) and α_2_δ-1^KO/KO^ (red bars) mice for Ca_V_2.2_HA (*I*; *n* = 72 and 54 ROIs, respectively), CGRP (*J*; *n* = 24 and 18 ROIs, respectively), IB4 (*K*; *n* = 24 and 18 ROIs, respectively), vGlut2 (*L*; *n* = 36 and 36 ROIs, respectively), and Homer (*M*; *n* = 24 and 18 ROIs, respectively) in laminae I, IIo, Iii, III, and combined deeper layers IV and V. ***P < 0.001 (two-way ANOVA and Bonferroni’s post hoc test). Data represent mean ± SEM. Box and whisker versions of these plots are in *SI Appendix*, Fig. S7, and details are in *SI Appendix*, Table S2.

Next, we examined the distribution of Ca_V_2.2_HA in the spinal cord, and found strong immunoreactivity for the channel subunit in the dorsal horn (Fig. 4*A*). There was very little Ca_V_2.2_HA in the ventral horn (Fig. 4*A*), and no specific staining in Ca_V_2.2^WT/WT^ spinal cord (Fig. 4 *A*, *i*). Taking regions of interest (ROIs) perpendicular to the pial layer (Fig. 4 *A*, *ii*), we found that within the dorsal horn, Ca_V_2.2_HA was most abundant in superficial laminae I and II (Fig. 4*B*). Here Ca_V_2.2_HA shares topographic distribution with both the presynaptic markers CGRP, which is present in peptidergic primary afferent terminals in laminae I and II-outer (Fig. 4*C* and *SI Appendix*, Fig. S3*A*), and with IB4, which is present in nonpeptidergic terminals, mainly in lamina II-inner (Fig. 4*D* and *SI Appendix*, Fig. S3*B*). Ca_V_2.2_HA was also associated with a postsynaptic marker of excitatory synapses, Homer (Fig. 4*E*).

### Ablation of α_2_δ-1 Reduces Ca_V_2.2_HA in the Dorsal Horn Without Effect on Other Synaptic Markers.

The distribution of Ca_V_2.2_HA in the dorsal horn was markedly reduced in α_2_δ-1^KO/KO^ mice (Fig. 4 *F*–*H*), particularly in the superficial layers (Fig. 4*I*). Following subtraction of nonspecific signal found in wild-type Ca_V_2.2 sections (Fig. 4*B*), the reduction in Ca_V_2.2_HA was 72.7, 65.9, 64.6, and 44.7% in layers I, II-outer, II-inner, and III, respectively (Fig. 4*I*). This decrease provides clear evidence for the essential role of α_2_δ-1 for Ca_V_2.2 trafficking to the primary afferent presynaptic terminals. In contrast, in the deeper layers of the dorsal horn (laminae IV and V), there was no effect of the ablation of α_2_δ-1 on the low level of Ca_V_2.2_HA present (Fig. 4*I*).

Next, we investigated whether the α_2_δ-1–mediated loss of Ca_V_2.2_HA in the dorsal horn was concomitant with a reduction in density or distribution of synaptic markers, since α_2_δ-1 has also been implicated in synaptogenesis (26). In contrast to the marked reduction in Ca_V_2.2_HA in the absence of α_2_δ-1 (Fig. 4*I*), there was no effect of α_2_δ-1 ablation on the overall immunostaining intensity or distribution in the dorsal horn of three primary afferent presynaptic markers, CGRP (Fig. 4*J*), IB4 (Fig. 4*K*), and vesicular glutamate transporter-2 (vGlut2) (Fig. 4*L*), and no effect on postsynaptic Homer immunostaining (Fig. 4*M*).

### Dorsal Rhizotomy Reduces Ca_V_2.2_HA in the Dorsal Horn of the Spinal Cord.

**Fig. 5.**
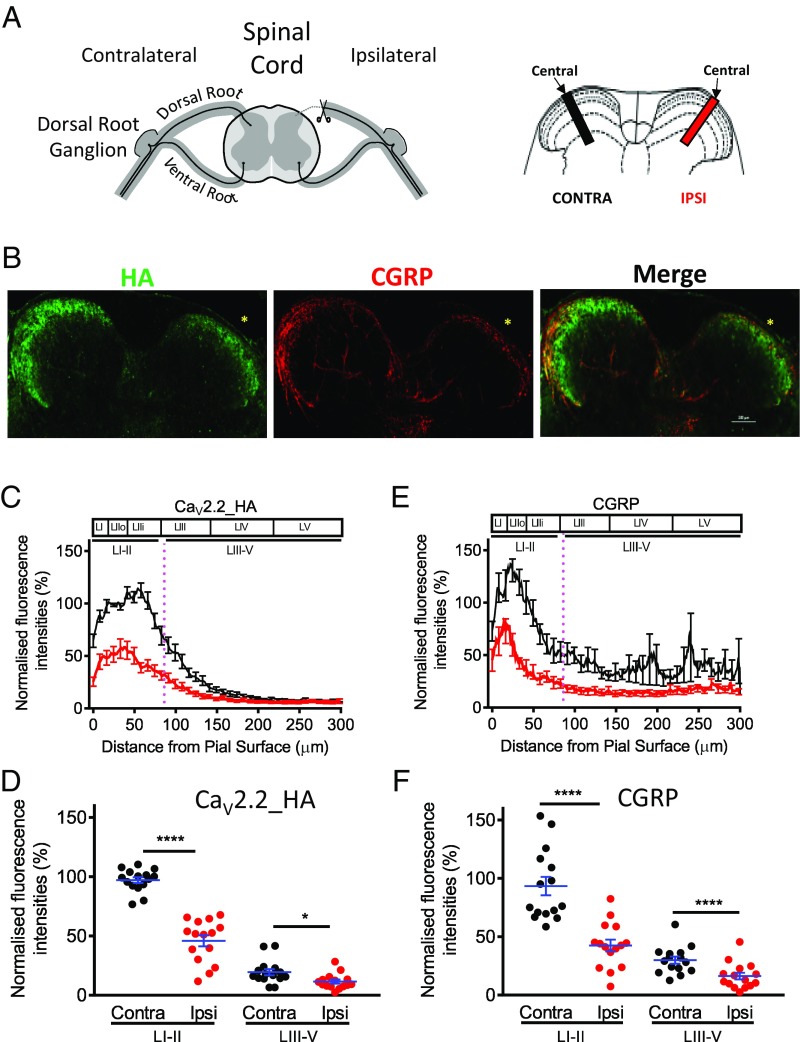
Effect of dorsal rhizotomy on Ca_V_2.2_HA distribution in the dorsal horn. (*A*) Diagram of the position of dorsal rhizotomy (*Left*) and central ROIs in the dorsal horn, ipsilateral (ipsi; red) and contralateral (contra; black) to rhizotomy (*Right*). (*B*) Images for Ca_V_2.2_HA (green; *Left*) and CGRP (red; *Middle*) from a Ca_V_2.2_HA^KI/KI^ mouse following rhizotomy (asterisks). Merged image (*Right*). (*C*) Plot profile of Ca_V_2.2_HA fluorescence intensity (mean ± SEM of 15 sections, normalized to the average contralateral intensity between 10 and 50 μm) in dorsal horn ROIs, contralateral (black) and ipsilateral (red) to rhizotomy. (*D*) Scatter plots of Ca_V_2.2_HA intensity (with blue mean ± SEM) for data from *C*, in superficial laminae I and II and in laminae III to V, contralateral (black circles) and ipsilateral (red circles) to rhizotomy. ****P < 0.0001, *P = 0.014 (paired *t* test). (*E*) Plot profile of CGRP intensity (mean ± SEM of 15 sections, normalized to the average contralateral intensity between 4 and 24 μm) in dorsal horn ROIs, contralateral (black line) and ipsilateral (red line) to rhizotomy. (*F*) Scatter plots of CGRP intensity (with blue mean ± SEM) for data from *E*, in superficial laminae I and II and in laminae III to V, contralateral (black circles) and ipsilateral (red circles) to rhizotomy. ****P < 0.0001 (paired *t* test).

In light of the marked reduction in Ca_V_2.2_HA, without loss of synaptic markers, in the dorsal horn of α_2_δ-1^KO/KO^ mice (Fig. 4*I*), we wished to examine further the extent of its origin in presynaptic primary afferent terminals. To investigate this, we performed unilateral dorsal rhizotomy (Fig. 5*A*). This resulted in a significant reduction of Ca_V_2.2_HA in the ipsilateral dorsal horn (Fig. 5 *B*–*D*). In the central ROI, the reduction was 52.7% in the superficial layers I and II, and there was also a substantial depletion (by 44.7%) in layers III to V (Fig. 5*D*). Rhizotomy is generally found to be incomplete, as longitudinal fibers remain intact (27). To determine the extent of the rhizotomy, we also examined the level of CGRP, as a marker of loss of presynaptic peptidergic afferents (27). A very similar extensive reduction of CGRP was observed, by 53.1% in layers I and II and 58.6% in layers III to V (Fig. 5 *E* and *F*). The correspondence between the reduction of Ca_V_2.2_HA and that of CGRP, whose origin is entirely presynaptic in the dorsal horn, confirms the mainly presynaptic localization of the Ca_V_2.2_HA signal in this region. Following dorsal rhizotomy, there was also a 20.7% decrease of α_2_δ-1 in central laminae I and II (*SI Appendix*, Fig. S4), which is expressed both in primary afferents and in intrinsic neurons (20). In contrast, there is no reduction in the NPY signal in the same region (*SI Appendix*, Fig. S4), this peptide being expressed mainly by dorsal horn interneurons (for a review, see ref. 28).

### Ca_V_2.2_HA Subcellular Localization in the Spinal Cord: Effect of α_2_δ-1 Ablation.

At higher resolution, we observed that Ca_V_2.2_HA, present in the superficial dorsal horn laminae, was distributed in rosette structures consisting of Ca_V_2.2_HA puncta surrounding a central core containing vGlut2 and often (but not always) associated with either CGRP (*SI Appendix*, Fig. S5 *A* and *B*) or IB4 (*SI Appendix*, Fig. S5 *C* and *D*), resembling glomerular synapses (29).

**Fig. 6.**
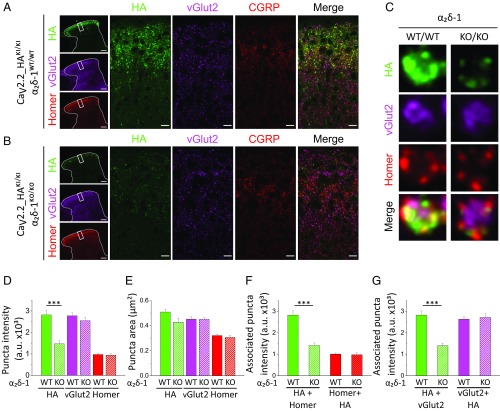
High-resolution analysis of Ca_V_2.2_HA puncta in the dorsal horn. (*A* and *B*) Airyscan images from two stitched tiles of 75 × 75 µm across the dorsal horn ROIs (on low-magnification images; *Left*), for Ca_V_2.2_HA (green), vGlut2 (magenta), Homer (red), and merged, from Ca_V_2.2_HA^KI/KI^ α_2_δ-1^WT/WT^ (*A*) and Ca_V_2.2_HA^KI/KI^ α_2_δ-1^KO/KO^ (*B*) sections. (Scale bars: 100 µm and 10 µm in low- and high-magnification images, respectively.) (*C*) Airyscan images (2 × 2 µm) of individual rosette clusters of Ca_V_2.2_HA puncta from Ca_V_2.2_HA^KI/KI^ α_2_δ-1^WT/WT^ (*Left*) and Ca_V_2.2_HA^KI/KI^ α_2_δ-1^KO/KO^ (*Right*) sections. Images (*Top* to *Bottom*) show Ca_V_2.2_HA (green), vGlut2 (magenta), Homer (red), and merged. (*D* and *E*) Puncta intensity (*D*) and puncta size (*E*) for Ca_V_2.2_HA^KI/KI^ α_2_δ-1^WT/WT^ (solid bars) and Ca_V_2.2_HA^KI/KI^ α_2_δ-1^KO/KO^ (hatched bars) for Ca_V_2.2_HA (green bars), vGlut2 (magenta bars), and Homer (red bars). ***P = 0.0014 (Student’s *t* test). (*F*) Associated puncta intensity for HA associated with Homer (green bars), and Homer associated with HA (red bars), for Ca_V_2.2_HA^KI/KI^ α_2_δ-1^WT/WT^ (solid bars) and Ca_V_2.2_HA^KI/KI^ α_2_δ-1^KO/KO^ (hatched bars). ***P = 0.0006 (Student’s *t* test). (*G*) Associated puncta intensity for HA associated with vGlut2 (green bars), and vGlut2 associated with HA (magenta bars), for Ca_V_2.2_HA^KI/KI^ α_2_δ-1^WT/WT^ (solid bars) and Ca_V_2.2_HA^KI/KI^ α_2_δ-1^KO/KO^ (hatched bars). ***P = 0.0008 (Student’s *t* test). All data are mean ± SEM of five Ca_V_2.2_HA^KI/KI^ α_2_δ-1^WT/WT^ and four Ca_V_2.2_HA^KI/KI^ α_2_δ-1^KO/KO^ ROIs, for 505 and 380; 1,264 and 1,153; or 1,021 and 731 HA and vGlut2 or Homer puncta for α_2_δ-1^WT/WT^ and α_2_δ-1^KO/KO^, respectively. Box and whisker versions of plots *D*–*G* are in *SI Appendix*, Fig. S8.

To improve resolution of these structures, we then obtained superresolution Airyscan images of Ca_V_2.2_HA together with vGlut2 and Homer in regions of the dorsal horn in both α_2_δ-1^WT/WT^ (Fig. 6*A*) and α_2_δ-1^KO/KO^ mice (Fig. 6*B*). The rosette-shaped clusters of Ca_V_2.2_HA consisted of groups of four or five puncta (Fig. 6*C*). These puncta may each correspond to individual active zones of primary afferent terminal glomerular synapses, because they are usually organized around a central core containing vGlut2, and also frequently apposed to the postsynaptic marker Homer (Fig. 6*C*).

We found the density of Ca_V_2.2_HA was markedly reduced in α_2_δ-1^KO/KO^ dorsal horn (Fig. 6 *B* and *C*), and we quantified the effect on several parameters associated with Ca_V_2.2_HA puncta (for a method, see *SI Appendix*, Fig. S6). The density of Ca_V_2.2_HA was reduced in individual clusters of puncta in α_2_δ-1^KO/KO^ dorsal horn, by 47.7% (Fig. 6*D*), but the cluster areas were not significantly affected (Fig. 6*E*). In contrast, neither the area nor the intensity of vGlut2 or Homer clusters was affected by loss of α_2_δ-1 (Fig. 6 *D* and *E*). In estimating the pairwise association between Ca_V_2.2_HA and Homer (Fig. 6*F*), or Ca_V_2.2_HA and vGlut2 (Fig. 6*G*), we found that the intensity of vGlut2 and Homer in these associated clusters was not affected in α_2_δ-1^KO/KO^ dorsal horn (Fig. 6 *F* and *G*). However, as expected, the intensity of Ca_V_2.2_HA in the associated clusters was reduced by 50.0% for Ca_V_2.2_HA puncta overlapping with Homer (Fig. 6*F*), and by 50.7% for those overlapping with vGlut2 (Fig. 6*G*).

### Subcellular Localization of Ca_V_2.2_HA.

**Fig. 7.**
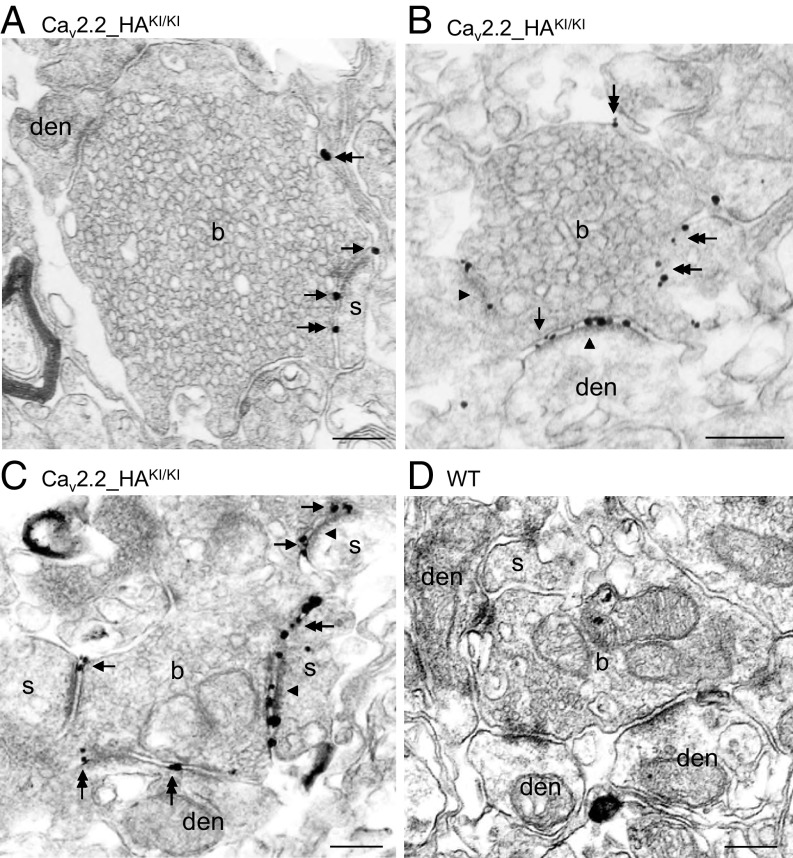
Subcellular distribution of presynaptic Ca_V_2.2 channels in putative primary afferent terminals in the dorsal horn of the spinal cord. (*A*–*C*) Electron micrographs showing immunoreactivity for Ca_V_2.2_HA in Ca_V_2.2_HA^KI/KI^ mice detected by the preembedding immunogold method. Immunoparticles labeling HA were observed in the active zone (arrowheads in *B* and *C*) of boutons (b), as well as localized to the perisynaptic (arrows) and extrasynaptic (double arrows) membrane segments of axon terminals making asymmetrical synapses with postsynaptic dendritic shafts (den) and dendritic spines (s). (*D*) No immunolabeling for the channel subunit was detected in tissues from control WT mice. (Scale bars: 200 nm.) Data are from *n* = 2 mice of each genotype.

To determine the subcellular localization of the Ca_V_2.2_HA channels, we used preembedding immunogold labeling. For electron microscopic investigation, tissue blocks were taken from the dorsal horn of the spinal cord. Immunoreactivity for Ca_V_2.2_HA was predominantly found in presynaptic elements, namely on axon terminals of presumed primary afferents (Fig. 7 *A*–*C*). Single or small clusters of immunogold particles were mainly localized to the active zone of boutons, including multiple active zones on individual glomerular boutons (Fig. 7 *B* and *C*), and also appeared at the edge of presynaptic membrane specializations (Fig. 7 *A*–*C*) and along the extrasynaptic plasma membrane (Fig. 7 *A*–*C*) of axon terminals making asymmetrical putative glutamatergic synapses with dendritic shafts and spines of postsynaptic neurons. The specificity of the immunolabeling was confirmed by the absence of immunoreactivity for Ca_V_2.2_HA in tissues obtained from control animals (Fig. 7*D*).

## Discussion

In this study, we have been able to visualize native N-type Ca_V_2.2 channels on the cell surface of neurons in vivo. We have concentrated here on the primary afferent neuronal pathway, because of the importance of Ca_V_2.2 in synaptic transmission in this system and its therapeutic importance as a drug target (7, 30). We show that Ca_V_2.2_HA is very strongly expressed on the cell surface, particularly of CGRP-positive small DRG neurons, and this is recapitulated in DRG neurons in culture. In contrast, transcriptional profiling found *Cacna1b* mRNA to be present in similar amounts in IB4-positive and IB4-negative nociceptors, the latter group including CGRP-positive DRG neurons (31). This would agree with the high intracellular Ca_V_2.2_HA we found in both CGRP-positive and CGRP-negative small DRG neurons. The localization of Ca_V_2.2_HA in DRG neurons is paralleled by striking expression of Ca_V_2.2_HA in the dorsal horn of the spinal cord, predominantly in laminae I and II. Here the presynaptic Ca_V_2.2_HA puncta are associated with the primary afferent markers CGRP, vGlut2, and IB4, present in glomerular primary afferent presynaptic terminals as described previously (29). The Ca_V_2.2_HA puncta are also adjacent to puncta containing the postsynaptic density protein Homer. The presynaptic localization of Ca_V_2.2_HA in primary afferents is confirmed through their ablation by dorsal rhizotomy. Furthermore, from the high-resolution immunoelectron-microscopic localization of Ca_V_2.2_HA, we confirm that these rosette structures formed by the Ca_V_2.2_HA puncta are likely to represent Ca_V_2.2_HA in active zones of individual glomerular terminals.

The α_2_δ-1 auxiliary subunit has been shown to be important for calcium channel trafficking in expression systems (13). It plays a major role in pain pathways and is up-regulated following neuropathic injury (17–20, 23). Furthermore, knockout of α_2_δ-1 caused a marked delay in the development of neuropathic mechanical hypersensitivity (24), and overexpression of α_2_δ-1 mimics features of neuropathic injury (23). In rats, α_2_δ-1 is expressed in all DRG neurons with highest expression in small neurons (20), and this distribution is confirmed here, in mice. However, until now it has not been possible to examine the effect of α_2_δ-1 on the trafficking of the relevant endogenous N-type channels in vivo.

Our results using Ca_V_2.2_HA^KI/KI^ mice crossed with α_2_δ-1^KO/KO^ mice, in which α_2_δ-1 is globally ablated, highlight the essential role of α_2_δ-1 in directing Ca_V_2.2_HA to the cell surface in DRG neurons and in targeting Ca_V_2.2_HA to presynaptic terminals in the dorsal horn. Accompanying the complete loss of DRG neuronal cell-surface Ca_V_2.2_HA, there was also a significant increase in cytoplasmic Ca_V_2.2_HA in CGRP-positive α_2_δ-1^KO/KO^ DRG neurons, indicating a defect in cell-surface trafficking.

The calcium currents in DRG neuronal somata in culture are found to be composed of between 20 and 50% N-type current, depending on the species, developmental stage, culture conditions, and subtype of DRG neuron examined (24, 32–35). One comprehensive study showed the proportion of N-type current was about 40% in cultured mouse DRG neurons with a diameter of less than 30 µm, and 20% in those larger than 30 µm (35), which is in agreement with the differential distribution of Ca_V_2.2_HA found here in small DRG neurons. We found previously that in cultured DRG neurons from α_2_δ-1 knockout mice the calcium channel current was only reduced by about 30% compared with wild-type DRG neurons, and the N-type current was reduced proportionately (24), which is in contrast to the marked effects of α_2_δ-1 knockout on Ca_V_2.2_HA localization described here. It is highly likely that even short-term cultured DRG neurons do not fully represent the in vivo situation, and that rapid changes occur in cell-surface expression of receptors and channels when cells are enzymatically dissociated and maintained in culture, allowing neurite outgrowth (36). Since evoked synaptic currents in laminae I and II are 74% N-type (37), there is likely to be a differential synaptic localization of these channels in vivo.

It has been found that there are other synaptic roles for α_2_δ-subunits unrelated to calcium channel function; for example, an association of the extreme C terminus of α_2_δ-1 with NMDA receptors has been identified (38). Furthermore, postsynaptic α_2_δ-1 has been implicated in central neurons as the binding partner of thrombospondins to promote synaptogenesis induced by this secreted protein family, independent of its role as a calcium channel subunit (26, 39). Thrombospondins alone promote the formation of silent synapses, lacking postsynaptic elements (40). However, we did not detect robust binding of thrombospondin-4 to α_2_δ-1 (41). By contrast, in cultured hippocampal neurons, neuroligin was also identified as a binding partner of thrombospondins mediating an increase in the rate of synaptogenesis (42).

Both presynaptic α_2_δ-3 (43) and α_2_δ-4 (44) have also been implicated in determining synaptic morphology in the auditory system and retina, respectively, although in these cases the synaptic abnormalities resulting from knockout of the respective α_2_δ-subunits are likely related to calcium channel dysfunction. In the present study, despite the effect of global ablation of α_2_δ-1, which strongly disrupted Ca_V_2.2_HA cell-surface localization, particularly of CGRP-positive small DRG neurons, and markedly reduced presynaptic terminal localization of Ca_V_2.2_HA in the dorsal horn of the spinal cord, we did not observe any reduction in other presynaptic markers of these primary afferents, CGRP, vGlut2, and IB4, or the postsynaptic marker, Homer. At the level of individual synapses, we did not find a reduction in area of Ca_V_2.2_HA–positive puncta clusters, but there was a very clear reduction in intensity of Ca_V_2.2 in each cluster, in the absence of α_2_δ-1. This result suggests that, if these puncta represent presynaptic active zones in primary afferent glomerular synapses, α_2_δ-1 has not affected the density of synapses in the dorsal horn, despite a large reduction in presynaptic Ca_V_2.2_HA intensity. However, whether there are changes in synaptic morphology will require more detailed examination at the EM level in the future.

## Methods

### Generation of Ca_V_2.2_HA Epitope-Tagged Knockin Mice.

The Ca_V_2.2_HA mouse line was generated by Taconic Artemis in the C57BL/6 background by homologous recombination with the targeting vector, which included the genomic region around exon 13 of the *Cacna1b* gene from clones of a C57BL/6J RPCIB-731 BAC library into which the sequence coding for the 2× HA tag was cloned. The targeting vector also carried the puromycin resistance gene (PuroR) as a positive-selection marker in intron 13 between two Flipper recombination sites and the negative-selection marker thymidine kinase outside the homologous regions. The targeting vector was linearized and transfected into embryonic stem cells. The homologous recombinant clones were isolated by positive and negative selection and injected into blastocysts from BALB/c. Highly chimeric mice were crossed with C57BL/6, and transmission to the germ line was confirmed by black offspring. The positive selective marker was removed by Flipper recombinase after crossing the first generation of knockin mice with Flp deleter transgenic mice. Subsequent backcrossing with wild-type C57BL/6 mice allowed us to select mice without the Flipper transgene and only the 2× HA tag insertion in exon 13. Genotyping PCR was performed with the primers forward, 5′-CACACCAGCATACATGCTCG-3′ and reverse, 5′-TCCAGCCTCACATGCTGC-3′, that bind to the intronic sequences just before and after exon 13 to generate amplicons of 279 and 345 for the wild-type and knockin allele, respectively. The Ca_V_2.2_HA^KI/KI^ mice showed no difference compared with Ca_V_2.2^WT/WT^ mice with respect to body weights (*SI Appendix*, Table S1).

The α_2_δ-1 knockout C57BL/6 mouse line described previously (24, 45) was crossed with the Ca_v_2.2_HA knockin mice to generate double-transgenic Ca_v_2.2_HA^KI/KI^ α_2_δ-1^KO/KO^ mice. It should be noted that male α_2_δ-1 knockout mice on a different genetic background showed a susceptibility to diabetes (46), although we have not noted excessive urination up to 11 wk of age in male double-transgenic mice. Both male and female mice were used in the present study. There was a small reduction of body weight in Ca_v_2.2_HA^KI/KI^ α_2_δ-1^KO/KO^ compared with Ca_v_2.2_HA^KI/KI^ α_2_δ-1^WT/WT^ mice for both sexes (*SI Appendix*, Table S1).

Mice were housed in groups of no more than five on a 12-h:12-h light:dark cycle; food and water were available ad libitum. All experimental procedures were covered by UK Home Office licenses, had local ethical approval by University College London (UCL) Bloomsbury Animal Welfare and Ethical Review Body, and followed the guidelines of the International Association for the Study of Pain (47).

### Additional Methods.

Methods for quantitative PCR, synaptosome preparation, immunoblotting, DRG neuronal cultures, electrophysiology, immunocytochemistry in cultured DRG neurons, dorsal rhizotomy, immunohistochemistry, confocal image acquisition and analysis, and preembedding immunoelectron microscopy are included in *SI Appendix*.

### Statistical Analysis.

Data were analyzed with Prism 5.0 or 7.0 (GraphPad Software) or OriginPro 2015 (OriginLab). Where error bars are shown, they are SEM; “*n*” refers to the number of cells or sections, unless indicated otherwise. Statistical significance between two groups was assessed by Student’s *t* test or paired *t* test, as stated. One-way ANOVA and stated post hoc analysis were used for comparison of means between three or more groups. All box and whisker plots show box (25 to 75%) and whisker (10 to 90%) plots with median (line) and mean (+).

## Supplementary Material

Supplementary File
