# Supplementary material for: Ablation of α2δ-1 inhibits cell-surface trafficking of endogenous N-type calcium channels in the pain pathway in vivo
Source: Proc Natl Acad Sci U S A. 2018 Nov 28;115(51):E12043–52. doi: 10.1073/pnas.1811212115 (PMC6305000; doi:10.1073/pnas.1811212115)
Supplement: Supplementary File [file pnas.1811212115.sapp.pdf]

Supplementary Information for

Visualizing endogenous N-type calcium channels in the pain pathway in vivo: $\alpha_2\delta$-1 ablation abolishes their cell surface trafficking

Manuela Nieto-Rostro, Krishma Ramgoolam, Wendy S Pratt, Akos Kulik and Annette C Dolphin

Corresponding author: Annette C. Dolphin (a.dolphin@ucl.ac.uk), Department of Neuroscience, Physiology and Pharmacology, University College London, Gower Street, London, WC1E 6BT, UK
Email: a.dolphin@ucl.ac.uk

This PDF file includes:

Supplementary Methods
Figs. S1 to S8
Tables S1 to S2

www.pnas.org/cgi/doi/10.1073/pnas.1811212115

SUPPLEMENTARY INFORMATION APPENDIX

SUPPLEMENTARY METHODS

Quantitative PCR

$Ca_V2.2\_HA^{KI/KI}$ and $Ca_V2.2\_HA^{WT/WT}$ mice (either 2 or 10 weeks old) were euthanized by CO_2 exposure and decapitated, according to Schedule 1 guidelines (Home Office Animals (Scientific procedures) Act 1986, UK). Brains were dissected as described previously [1]. Tissue samples were disrupted using a rotor-stator homogenizer (Disperser T10, IKA, Staufen, Germany). Total RNA was extracted from one hemisphere of homogenised brain tissue using the RNeasy Lipid Tissue Kit (Qiagen) including an on-column DNase step. Reverse transcription was performed on 5µg of RNA carried out using High capacity RNA-to-cDNA kit (Applied Biosystems). TaqMan qRT-PCR (40 cycles) of sample triplicates was used to determine the relative abundance of the $Ca_V2.2$ α1 subunit, performed with an Applied Biosystems 7500/7500 Fast Real-Time PCR system. The following TaqMan assays with TaqMan Gene Expression Master Mix were used in accordance to the manufacturer's protocol (gene name: assay ID): Hypoxanthine phosphoribosyltransferase (*Hprt1)*: Mm00446968_m1; *Cacna1b*: Mm01333678_m1; Glyceraldehyde 3-phosphate dehydrogenase (*Gapdh)*: Mm99999915_g1. The comparative C_T ($\Delta\Delta C_T$) method was used for relative quantification of fold differences (given as the mean ± s.e.m.) of $Ca_V2.2$ mRNA levels in $Ca_V2.2\_HA^{KI/KI}$ and $Ca_V2.2\_HA^{WT/WT}$ mice at 2 and 10 weeks of age. *Hprt1* was determined as the most stably expressed reference gene and data were normalised for expression of *Hprt1* mRNA by calculating ΔC_T. Measurements were performed on at least three independent RNA preparations from each age.

Synaptosome preparation

Synaptosomal fractions were prepared by differential centrifugation [2, 3]. Spinal cord from $Ca_V2.2\_HA^{KI/KI}$ and $Ca_V2.2\_HA^{WT/WT}$ mice were homogenized in buffer I (0.32M sucrose, 3mM HEPES-Na, pH 7.4, 0.25mM DTT containing protease cocktail inhibitor (Roche Diagnostics, Lewes, UK)). The homogenate was centrifuged at 1,000 x g for 10 min at 4°C to produce a pellet (P1) and a supernatant (S1). The pellet P1 was resuspended in buffer I and centrifuged at 1,000 x g for 10 min at 4°C. This produced a pellet (P1') and a supernatant (S1'). S1' and S1 were combined and centrifuged at 12,000 x g for 15 min to produce pellet P2 and supernatant S2. P2 was resuspended in buffer I and centrifuged for 15 min at 13,000 x g to yield the crude synaptosomal fraction (P2'). P2' was solubilized in 50mM Tris HCl pH7.4, 150mM NaCl, 0.5 % NaDeoxycholate, 0.1 % SDS, 1 % Igepal) for 45 min on ice. The solubilized mixture was clarified by centrifugation for 30 min at 12,000 x g. This produced a supernatant termed the synaptosomal fraction which was subject to immunoblotting.

Immunoblotting

60 μg per sample was loaded onto 3-8 % NuPage Tris/acetate gels (Invitrogen) and proteins were resolved by SDS-polyacrylamide gel electrophoresis (SDS-PAGE). Proteins were transferred to polyvinylidene difluoride (PVDF) membranes (Bio-Rad). After blocking (10mM Tris pH7.4, 500mM NaCl, 0.5 % Igepal, 3 % BSA) blots were probed with rat monoclonal anti-HA (1:500; Roche) and mouse monoclonal anti-GAPDH (1:25000; Ambion) antibodies at 4 ^{o}C overnight. The protein-antibody complexes were then labelled with a horseradish-peroxidase-conjugated secondary antibody (1:2000, Sigma) for 1 h at room temperature (RT). Bands were detected using the enhanced ECL Plus reagent (GE Healthcare) visualized with a Typhoon 9410 scanner (GE Healthcare).

DRG Neuronal Cultures

DRGs were removed from the entire spine of Ca_V2.2_HA$^{KI/KI}$ and Ca_V2.2_HA$^{WT/WT}$ mice at 10 weeks old. DRG neuronal cultures were obtained using similar methods described in (Hendrich et al., 2008) through enzymatic and mechanical dispersion. The DRG tissue was vigorously shaken for 18 min at 37°C in Hank's basal salt solution containing 1000U/ml DNAse 1 (Invitrogen), 3.75mg/ml dispase (Invitrogen) and 0.8mg/ml collagenase type 1A (Sigma). The partially digested tissue was washed and triturated in growth medium (DMEM/F12 with 10 % fetal bovine serum), 2mM GlutaMAX (Invitrogen) and 100 U penicillin-100 μg streptomycin/ml (Invitrogen). For immunocytochemistry experiments, DRG neurons were then plated on coverslips coated with poly-L-lysine (Sigma) and laminin (Sigma) in growth medium containing 0.5μl/ml nerve growth factor (NGF). For electrophysiological experiments, laminin was not used as a substrate when plating DRG neuronal cultures.

Electrophysiology

Calcium channel currents in DRG neurons were investigated by whole cell patch clamp recording (after 1 DIV). The patch pipette solution contained (in mM): 140 Cs-aspartate, 5 EGTA, 2 $MgCl_2$, 0.1 $CaCl_2$, 2 K_2ATP, 20 HEPES, pH 7.2, 310 mOsm with 3M CsOH. The external solution for recording Ba^{2+} currents contained (in mM): 150 tetraethyl-ammonium Br, 3 KCl, 1.0 $NaHCO_3$, 1.0 $MgCl_2$, 10 HEPES, 4 D-glucose, 2 $BaCl_2$, 0.001 TTX, pH 7.4, 320 mOsm with Sigma 7-9 tris-base buffer. Electrophysiology was performed using an Axopatch 200B amplifier with pClamp 10.2 (Molecular Devices). Pipettes of resistance 2 -4 MΩ were used. Data were filtered at 1 - 2 kHz and digitized at 5 - 10 kHz. To record current - voltage (IV) relationships, cells were held at

−80 mV, and a 100 ms step potential was applied to between −60 and +50 mV in 10 mV intervals every 10s. Analysis of data was performed using pClamp 10.7 and Origin Pro 2017 (Originlab). Mean I-V relationships were fitted with a modified Boltzmann equation: $I = G_{max} (V-V_{rev})/ (1+exp[-(V-V_{50,act})/k])$, where G_{max} is the maximum conductance, V_{rev} is the reversal potential, k is the slope factor and $V_{50,act}$ is the voltage for 50 % current activation.

Immunocytochemistry in cultured DRG neurons

Ca_V2.2_HA staining was investigated in DRG neurons by immunocytochemistry (after 1 DIV). For IB4 staining, DRG neuronal cultures were incubated with 1:100 IB4 conjugated to fluorescein isothiocyante (IB4-FITC; Sigma) for 10 min at 37°C, and then washed with Krebs Ringer Hepes (KRH) buffer. After this, cells were fixed with 4 % PFA and 4 % sucrose in PBS for 5 min and then blocked (PBS, 20 % goat serum, 4 % BSA) for 1 h at RT. To detect surface Ca_V2.2_HA staining, cells were incubated with rat anti-HA (1:200; Roche) overnight at 4°C. Cells were then incubated with anti-rat Alexa Fluor 594 secondary antibody (1:500; Invitrogen) for 1 h at RT. Following this, DRG neurons were permeabilized with 0.1 % Triton X-100 for 10 min; and to reduce the background signal resulting from the use of a mouse Ab on mouse DRG neuronal cultures, endogenous IgGs were blocked by incubation with the unconjugated Fab anti-mouse IgG (H+L) (0.1 mg/ml in PBS, Jackson ImmunoResearch Laboratories) for 1 h at RT. To detect CGRP, DRG neurons were incubated in mouse anti-CGRP (1:1000; Sigma) overnight at 4°C followed by anti-mouse Alexa Fluor 633 (1:500; Life Technologies) for 1 h at RT.

Staining with NF200 (rabbit polyclonal Ab, 1:200; Sigma) was performed (after surface Ca_V2.2_HA had been detected by the aforementioned antibodies) in permeabilized DRG neurons overnight at 4°C. Secondary antibody anti-rabbit Alexa Fluor 488 was used at 1:500 (Life Technologies) for 1h at RT. Nuclei were stained with DAPI (500 nM) before mounting on slides using Vectashield (Vector Laboratories) to reduce photobleaching. Imaging was performed using a confocal laser-scanning microscope (Zeiss) and a 63X oil immersion objective. Optical sections of 0.5 μm thickness were acquired for each channel.

DRG neurons that were judged as IB4-positive by the observer consistently had intensities ≥ 20 %, whereas those considered as IB4-negative consistently had intensities ≤20%. Therefore, the borderline between IB4 positive and IB4-negative in this study was taken as 20% intensity [4]. All data were normalized to the appropriate highest intensity.

Dorsal rhizotomy

Three male mice were anesthetised with 2.5 % isoflurane and the lumbar spinal cord exposed by laminectomy. Two (L4 & L5, n=1) or three (L3-L5, n=2) dorsal roots were sectioned on one side, midway between the DRG and the dorsal root entry zone. The surrounding muscle was closed with absorbable 6-0 vicryl sutures (Ethicon, VetTech, UK), and the skin closed with surgical wound clips. After surgery the mice were allowed to recover for 7-10 days, before perfusion fixation for immunohistochemistry.

Immunohistochemistry

For immunohistochemistry, mice were deeply anaesthetized with an intraperitoneal injection of pentobarbitone (Euthatal, Merial Animal Health, Harlow, UK; 600 mg / kg), perfused transcardially with saline containing heparin, followed by perfusion with 4 % paraformaldehyde in 0.1 M phosphate buffer (PB, pH 7.4) at a flow rate of 2.5 $ml.min^{-1}$ for 4 min. Lumbar 3 − 5 DRGs and the lumbar enlargement of the spinal cord were dissected out. Following dissection, the spinal cord was post-fixed for 2 h, whereas the DRGs did not undergo extra fixation. Tissue was washed with PB, cryoprotected by incubation in PB with 15 % sucrose overnight, and finally mounted in Optimal cutting temperature (OCT) compound (VWR) before storing at -80 ^{o}C until sectioning with a cryostat. DRGs and spinal cord were sectioned at 15 and 20 μm respectively using a cryostat, placing the sections sequentially in series of 6 slides, so the distance between any section and the next in any slide is 90 or 120 μm in each case. Slides were stored at -80 ^{o}C until processed.

For immunofluorescence labeling of DRGs, sections were blocked with 10 % goat serum in PBS containing 0.3 % Triton X-100 for more than 1 h at RT, followed by incubation with the unconjugated goat Fab anti-mouse IgG (H+L) (0.1 mg/ml in PBS, Jackson ImmunoResearch Lab) for 1 h at RT, washed in PBS, 0.1 % Triton X-100 (PBS-T) and then incubated with rat monoclonal anti-HA antibody (Roche, 1:200), and either rabbit anti-Calcitonin Gene Related Protein Ab (CGRP, Sigma, 1:1000) or rabbit anti-NF200 Ab (Sigma, 1:200) for 2-3 days at 4° C in 5 % goat serum, 0.3 % Triton X-100 in PBS. Following extensive washing in PBS-T, immunolabelled samples were fixed in 4 % paraformaldehyde in PBS for 30 min at RT, washed in PBS-T and incubated for 1-2 days at 4° C with the goat anti-rat and anti-rabbit Abs conjugated with Alexa Fluor 488 and Alexa Fluor 594 respectively (Invitrogen, both 1:500). After washing, sections were treated with the nuclear stain DAPI and mounted in VectaShield (Vector Laboratories). Some DRG sections were labelled for $\alpha_2\delta$-1 as described previously (Patel et al., 2013) with the following modifications: after heat-induced epitope retrieval (10 mM citrate buffer, pH 6.0, 0.05 % Tween 20, 95°C for 10 min), the sections were blocked with 10 % goat serum in PBS containing 0.3 % Triton and treated with the unconjugated goat Fab anti-mouse IgG (H+L) (0.1 mg/ml in PBS, Jackson ImmunoResearch Lab) for 1 h at RT. Mouse monoclonal anti

dihydropyridine receptor (α2-1 subunit) Ab (Sigma, 1:100) and rabbit anti-CGRP Ab (as above) were applied for 2 or 3 days at 4 ^{o}C. After extensive washes, the samples were incubated with biotin-conjugated goat anti-mouse Fab fragment (1:500, Jackson ImmunoResearch Lab) and goat anti rabbit Ab conjugated with Alexa Fluor 594, overnight at 4 ^{o}C, followed by washes and Streptavidin-AlexaFluor-488 overnight at 4 ^{o}C (both at 1:500, Invitrogen).

For spinal cord immunohistochemistry, sections were incubated with rat monoclonal antibody anti-HA (as above) and either rabbit anti-CGRP (as above), IB4 conjugated with FITC (Sigma) or rabbit anti-Homer1 (Frontier institute, 1:2000), co-staining in some cases with a guinea pig anti-vGlut2 Ab (Millipore, 1:5000). Following incubation with primary Abs for 2-3 days at 4°C, samples were post-fixed for 30 min at RT with 4 % paraformaldehyde in PBS. According to the primary antibody combination used, in each case the secondary antibodies were anti-rat Alexa Fluor 488 or 594, anti-rabbit Alexa Fluor 594 and anti-guinea pig Alexa Fluor 633 (all goat antibodies from Invitrogen). In some of the rhizotomy sections, rabbit anti-NPY (Abcam, 1:1000) or mouse monoclonal anti dihydropyridine receptor (α2-1 subunit) Ab (Sigma, 1:100) were used.

Confocal Image acquisition and analysis

Immunostaining was visualized using a LSM 780 (Zeiss) confocal microscope. Images were acquired with constant settings in each experiment from at least 3 sections per sample from at least three mice unless otherwise stated. Only sections that were intact, unfolded and with uniform staining were selected for imaging and analysis. For DRG sections, multiple images were acquired with a 63x 1.4NA objective (1 Airy Unit) covering the whole area of the section containing neurons, and stitched with Zen software (Zeiss). For analysis, using ImageJ software (Schneider et al., 2012), in each and every intact DRG neuron with a visible nucleus we selected 2 different types of regions of interest (ROI) for the HA image. Using images with temporarily enhanced brightness and contrast, solely to aid visualization of the circumference of even dimly-stained cells, first we drew a 10 pixel wide line (0.9µm) following the perimeter of the cell from which we recorded the length as an estimation of the size of the cell (small <61 µm, medium 61-94 µm or large >94 µm) [5], and the mean membrane intensity. The line width was chosen for practical reasons to make sure that the membrane was covered, as it can be quite convoluted, but comparing a subset of measurements with a 5 µm line did not alter the results obtained. Secondly we selected an ROI for the area inside the first ROI, excluding the plasma membrane and the nucleus, to record the mean intracellular intensity. ROIs outside each section were used as background and deducted from sample measurements. In a second step of the analysis, the intracellular ROIs were placed in the CGRP or NF200 image and cells were categorized as CGRP- or NF200- positive or negative, based on levels of staining observed. Three different researchers

performed this analysis, in some cases blind, with very similar results, and data from different experiments were pooled for statistical analysis.

Spinal cord images at low magnification were acquired using a 20x 0.8 NA objective (5µm optical section) covering the whole section, and stitched with Zen software as for DRG sections. For analysis, using the same software, the mean intensity was recorded from a profile scan of a rectangular ROI of 50 x 300 µm placed across the superficial layers of the central part of each dorsal horn. The HA, CGRP, IB4, Homer and vGlut2 data from different experiments were pooled according to genotype and presented as the mean ± s.e.m. Similar analysis was performed to study the distribution of HA and other markers in spinal cord sections of mice that underwent rhizotomy, with 6 ROIs placed in the central part of the ipsi-lateral (ipsi) and contra-lateral (contra) dorsal horn of each section. All comparisons of $\alpha_2\delta$-1$^{WT/WT}$ and $\alpha_2\delta$-1$^{KO/KO}$ mice were from experiments performed in parallel (SI Appendix, Table S2).

For high magnification examination of spinal cord, a 63x 1.4 NA objective was used in either conventional confocal (0.8-0.7 µm optical sections) for HA, with CGRP and IB4 and vGlut2, or Airyscan mode (0.2 µm optical sections) for HA, Homer and vGlut2; super-resolution images then underwent pixel reassignment and Airyscan processing (7x) using Zen software. To quantify Ca_V2.2_HA density and its association with pre- and post-synaptic vGlut2 and Homer, a rectangular ROI across the superficial layers of the central dorsal horn was acquired with a tile of 2 Airyscan images of 75 x 75 µm and analyzed with FIJI software [6]. Each image was split into 3 channels and thresholded (auto triangle threshold) to create a mask per channel with the all the clusters between 50 and 2500 pixels (90 – 4500 nm^2) selected using the particle analyzer command. All the clusters were saved as list of ROIs and used in the original image to record their size and mean intensity. The corresponding masks for HA and vGlut2, or HA and Homer, were merged and the overlapping clusters (>1 %) were extracted using the plugin Binary Feature Extractor from the BioVoxxel Toolbox (http://www.biovoxxel.de), to obtain the associated clusters (SI Appendix, Fig. S6). 5 and 4 ROIs from different sections were analyzed in this way for one Ca_V2.2_HA$^{KI/KI}$ $\alpha_2\delta$-1$^{WT/WT}$ and one Ca_V2.2_HA$^{KI/KI}$ $\alpha_2\delta$-1$^{KO/KO}$ mouse respectively.

To better resolve CGRP- and IB4-positive glomeruli from a stack of conventional confocal images, ROIs of 2 x 2 µm were subject to deconvolution, with sequential use of ImageJ plugins “Diffraction PSF 3D” and “Iterative deconvolve 3D” per channel from ImageJ (http://www.optinav.info/Iterative-Deconvolve-3D.htm).

Pre-embedding immunoelectron microscopy.

Immunohistochemical labeling for electron microscopy was performed as described earlier [7]. Briefly, 10 week-old Ca_V2.2_HA$^{KI/KI}$ mice (n=2) and control wild-type mice (n=2) were used.

Mice were deeply anesthetized as above, and perfused transcardially. First, the vascular system was flushed by 0.9 % NaCl containing heparin for 1 min, followed by transcardial perfusion with a fixative prepared in 0.1 M PB containing 4 % paraformaldehyde, 15 % saturated picric acid and 0.05 % glutaraldehyde, for 13 min at a flow rate of 7.5 $ml.min^{-1}$. After perfusion, spinal cords were removed, sections were cut at 50 µm on a vibratome (VT1000, Leica, Wetzlar Germany), blocked and then incubated in rat monoclonal anti-HA antibody (4 mg/ml; Roche Diagnostics GmbH, Germany) diluted in tris-buffered saline (TBS) containing 3 % normal goat serum (NGS; Vector Laboratories,Burlingame, CA, USA) at 4°C overnight (O/N). Subsequently, they were incubated in goat anti-rat secondary antibody (Fab fragment, 1:100, Nanoprobes, Stony Brook, NY, USA) coupled to 1.4 nm gold particles at 4 °C O/N. Later, sections were processed for silver enhancement of the gold particles with an HQ Silver kit (Nanoprobes). Sections were then treated with 1 % osmium tetroxide and uranyl acetate, dehydrated, then embedded in epoxy resin (Durcupan ACM Fluka; Sigma-Aldrich, Gillingham, UK). Ultrathin sections were cut on an ultramicrotome (Reichert Ultracut E; Leica, Austria) and observed in a Zeiss LEO 906 E electron microscope.

REFERENCES

1. Schlick B, Flucher BE, Obermair GJ. Voltage-activated calcium channel expression profiles in mouse brain and cultured hippocampal neurons. *Neuroscience* **167**, 786-798 (2010).

2. Kato AS, *et al.* New transmembrane AMPA receptor regulatory protein isoform, gamma-7, differentially regulates AMPA receptors. *J Neurosci* **27**, 4969-4977 (2007).

3. Ferron L, Nieto-Rostro M, Cassidy JS, Dolphin AC. Fragile X mental retardation protein controls synaptic vesicle exocytosis by modulating N-type calcium channel density. *Nat Commun* **5**, 3628 (2014).

4. Fang X, *et al.* Intense isolectin-B4 binding in rat dorsal root ganglion neurons distinguishes C-fiber nociceptors with broad action potentials and high Nav1.9 expression. *J Neurosci* **26**, 7281-7292 (2006).

5. Sommer EW, Kazimierczak J, Droz B. Neuronal subpopulations in the dorsal root ganglion of the mouse as characterized by combination of ultrastructural and cytochemical features. *Brain Res* **346**, 310-326 (1985).

6. Schindelin J, *et al.* Fiji: an open-source platform for biological-image analysis. *Nat Methods* **9**, 676-682 (2012).

7. Booker SA, *et al.* KCTD12 Auxiliary Proteins Modulate Kinetics of GABAB Receptor-Mediated Inhibition in Cholecystokinin-Containing Interneurons. *Cereb Cortex* **27**, 2318-2334 (2017).

SUPPLEMENTARY FIGURES

Fig. S1. $Ca_V2.2$_HA cell surface expression on NF200-negative and positive DRG neurons

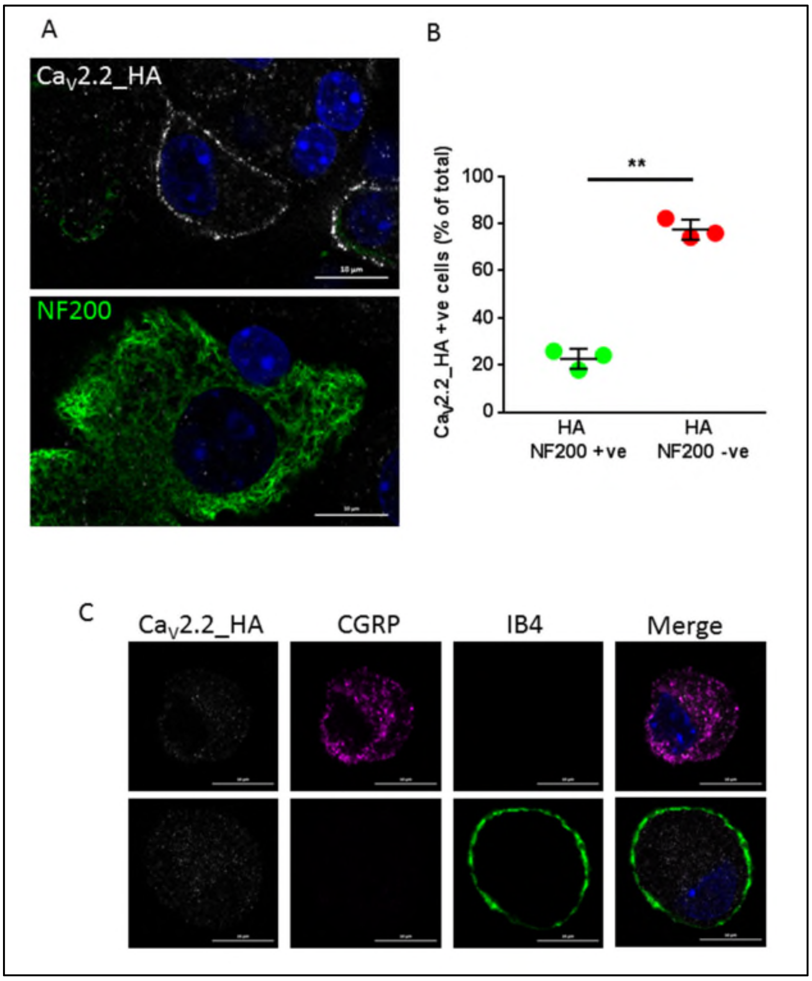

(A) Images of cultured DRG neurons showing $Ca_V2.2$_HA staining (white) prior to permeabilization, and NF200 staining (green) following permeabilization, for a representative NF200-negative (top) and NF200-positive (bottom) cell. DAPI nuclear staining is in blue.

(B) Quantification of percentage of DRG neurons with cell surface $Ca_V2.2$_HA that were also positive for NF200 (green circles), or negative for NF200 (red circles). Data are scatter plots (with superimposed mean ± s.e.m.) for 3 separate experiments and a total of 400 DRG neurons. Statistical difference was determined by paired t test, ** $P = 0.0079$.

(C) Images of cultured DRG neurons from WT mice, showing (left to right) lack of HA immunostaining staining prior to permeabilization, CGRP staining following permeabilization, IB4-FITC and merged image with DAPI (blue, staining the nuclei), for two representative CGRP-positive (top) and IB4-positive (bottom) cells.

Fig. S2. Intracellular α₂δ-1 and Ca$_V$2.2_HA staining quantified with respect to cell size

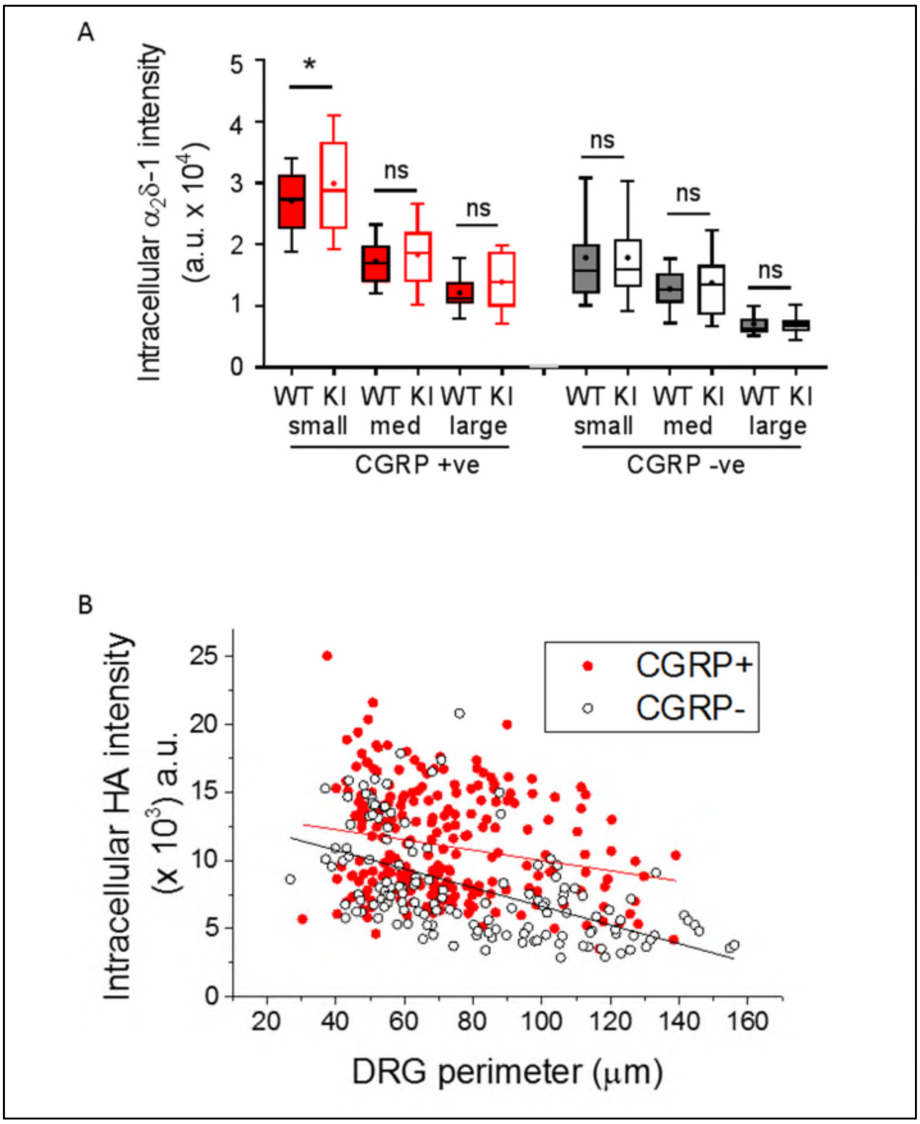

(A) Intracellular $\alpha_2\delta$-1 intensity for CGRP-positive (red bars) and CGRP-negative (black bars) DRG neurons from Ca$_V$2.2$^{WT/WT}$ $\alpha_2\delta$-1$^{WT/WT}$ (solid bars) and Ca$_V$2.2_HA$^{KI/KI}$ $\alpha_2\delta$-1$^{WT/WT}$ (open bars) mice. n = 73, 115 (small CGRP+); 90, 53 (med, CGRP+); 33, 8, (large, CGRP+); 69, 110 (small CGRP-); 152, 74 (med, CGRP-); 63, 25 (large, CGRP-) WT and KI DRG neurons, respectively, from 3 sections from two mice per genotype. Data are box (25-75 %) and whisker (10-90 %) plots with median (line) and mean (+). * P < 0.0.348, 1-way ANOVA and Bonferroni's multiple comparison test of selected pairs of columns. Statistical differences between $\alpha_2\delta$-1 levels in CGRP+ and CGRP – neurons are $P <$ 0.0001 (small WT, med WT and small KI); $P = 0.0007$ (med KI); $P = 0.0029$ (large WT); ns (large KI).

(B) Negative correlation between intracellular Ca$_V$2.2_HA and DRG neuronal cell size in $\alpha_2\delta$-1$^{KO/KO}$ DRGs. Intracellular Ca$_V$2.2_HA staining quantified with respect to DRG neuronal cell size, for CGRP-positive (solid red circles) and CGRP-negative (open black circles) DRG neurons from $\alpha_2\delta$-1$^{KO/KO}$ mice. Linear regression analysis for CGRP-positive data (red line; slope = -37.72, $r^2 = 0.0460$; DF = 230, F = 11.08, P = 0.0010) and CGRP-negative data (black line; slope = -68.6, $r^2 = 0.292$; DF = 142, F = 58.55, P < 0.0001).

Fig. S3. Comparison of distribution of Ca$_V$2.2_HA with CGRP and IB4 in dorsal horn

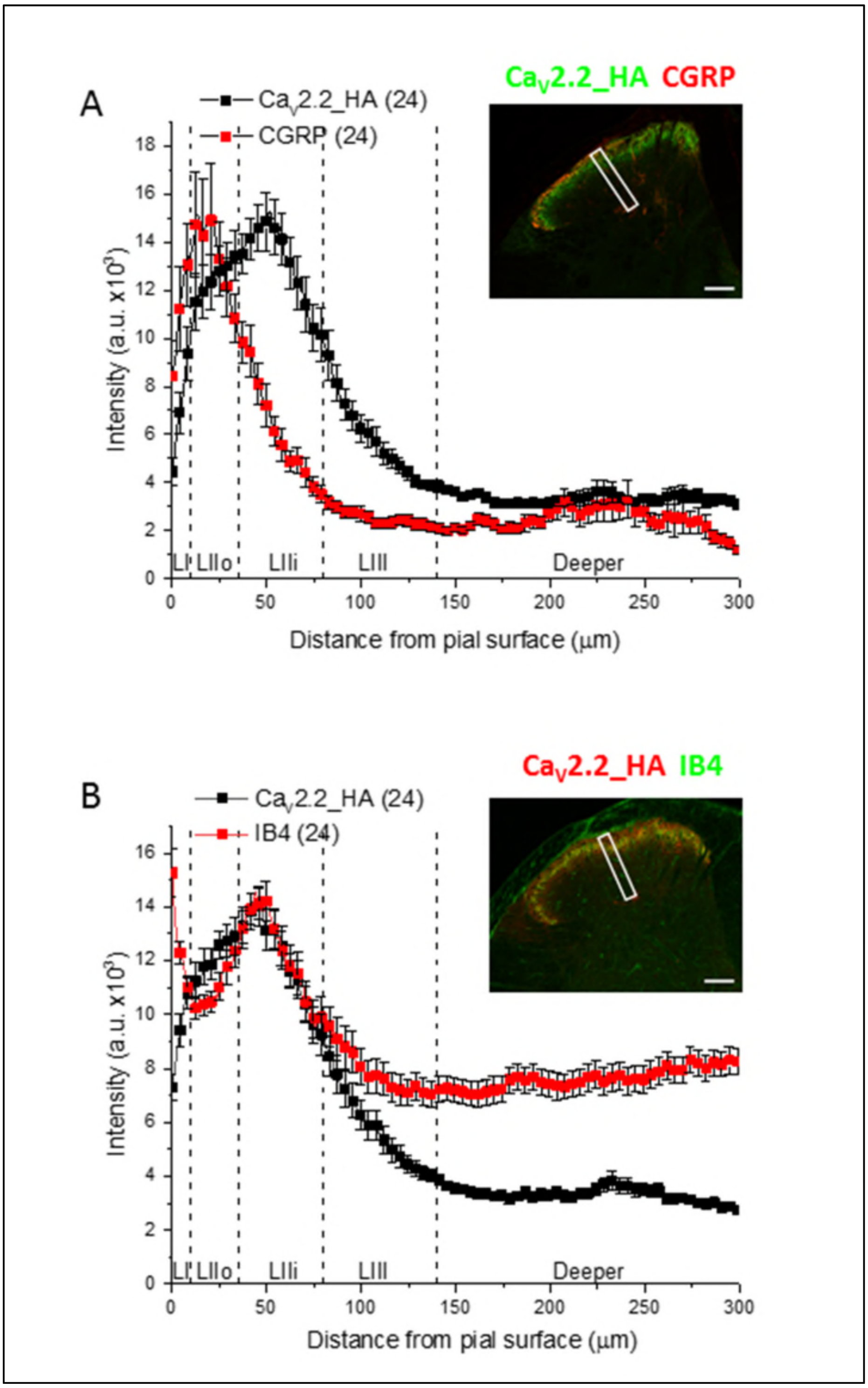

(**A, B**) Ca$_V$2.2_HA (black squares) and either CGRP (**A**) or IB4 (**B**) (red squares) fluorescence intensity plots (mean ± s.e.m.) from ROIs (50 x 300 µm) perpendicular to the pial surface and across the superficial laminae of the dorsal horn (Lamina I, LI: 1-10 µm, Lamina II outer, LIIo: 10-35 µm, Lamina II inner, LIIi: 35-80 µm, Lamina III, LIII: 80-140 µm, Deeper laminae: 140-300 µm) of central dorsal horn, from 24 ROIs (4 Ca$_V$2.2_HA$^{KI/KI}$ mice with 6 ROIs per mouse). Insets: Representative images of dorsal horn stained for HA and CGRP or HA and IB4, with ROIs depicted as white rectangles; scale bar 100 µm.

Fig. S4. Effect of dorsal rhizotomy on distribution of $\alpha_2\delta$-1 and NPY in dorsal horn central ROI

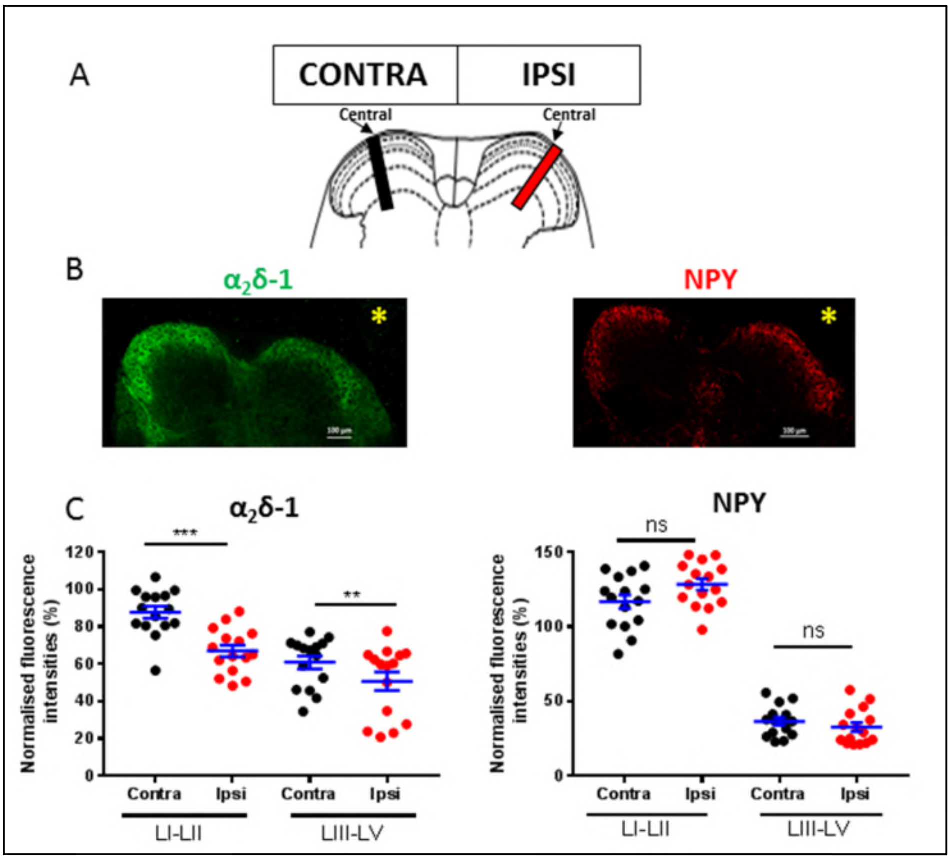

(A) Position of central ROIs in dorsal horn ipsilateral (ipsi, red) and contralateral (contra, black) to rhizotomy.

(B) Representative images for (left to right) $\alpha_2\delta$-1 and NPY, in dorsal horn from $Ca_V2.2$_HA$^{KI/KI}$ mouse following rhizotomy (surgery on right, yellow *).

(C) Fluorescence intensity for each panel ($\alpha_2\delta$-1, left; NPY, right) in superficial laminae I – II and in laminae III - V, contralateral (black circles, n = 15 sections) and ipsilateral (red circles, n = 15 sections) to the rhizotomy (with superimposed blue mean ± s.e.m.). *** $P = 0.0003$, ** $P = 0.007$, ns = not significant, paired t test.

Fig. S5 Localization of CGRP and IB4 in dorsal horn synapses, associated with $Ca_V2.2$_HA

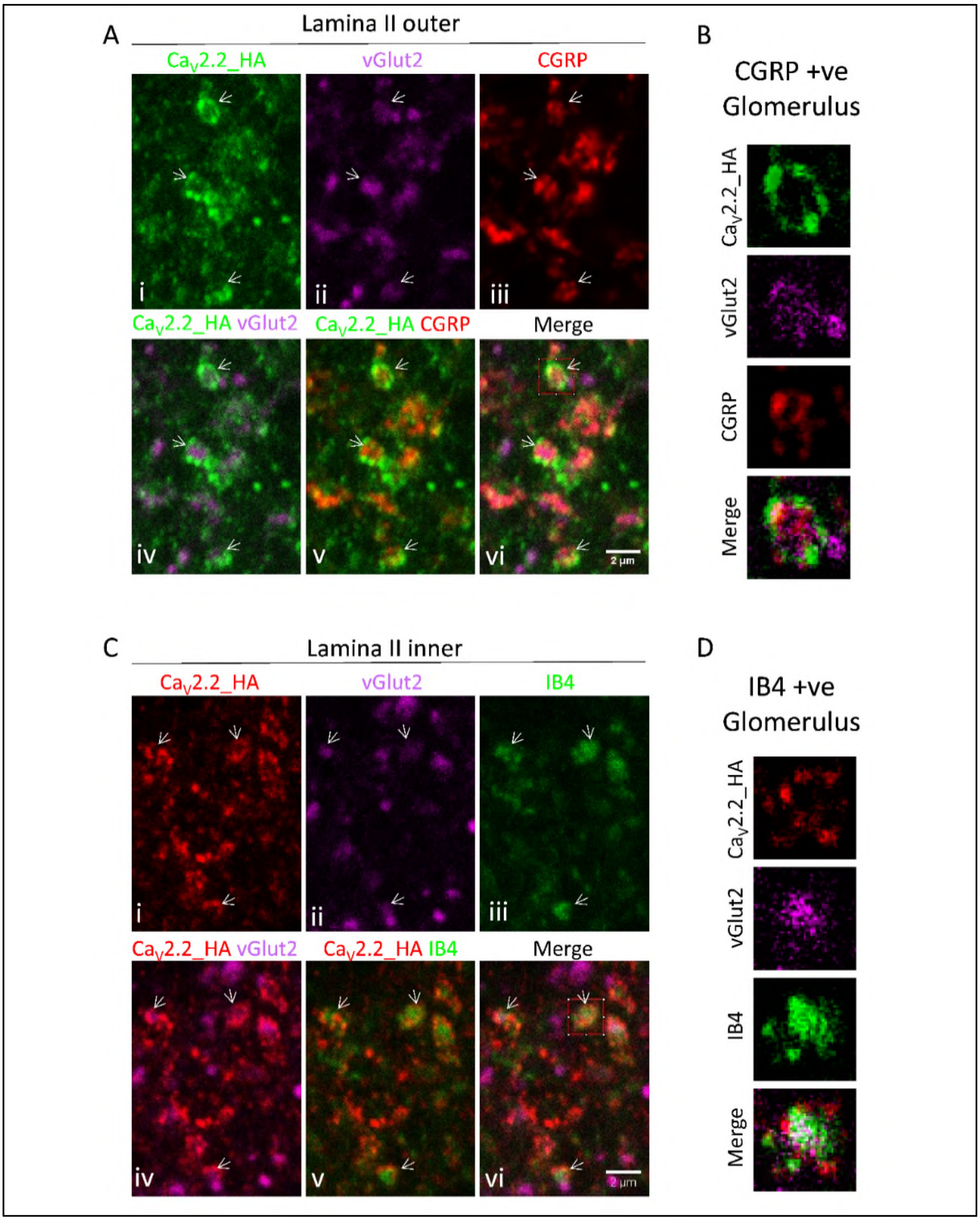

(**A, C**) High magnification images showing $Ca_V2.2$_HA puncta (i) in a rosette pattern (arrows) around vGlut2-positive boutons (ii), which are also positive for CGRP (**A**, iii) in Lamina II outer or IB4 (**C** ,iii) in Lamina II inner. (iv - vi) show merged images, and red ROIs marked in (vi) show images used in B and D. Scale bars 2 μm.

(**B, D**) Images (2 x 2 μm) of individual rosette clusters of $Ca_V2.2$_HA puncta, following deconvolution. Images (top to bottom) show $Ca_V2.2$_HA (green, **B**; red, **D**), vGlut2 (magenta), CGRP (red) or IB4 (green) and merged image.

Fig. S6. Cluster analysis from high resolution Airyscan confocal images

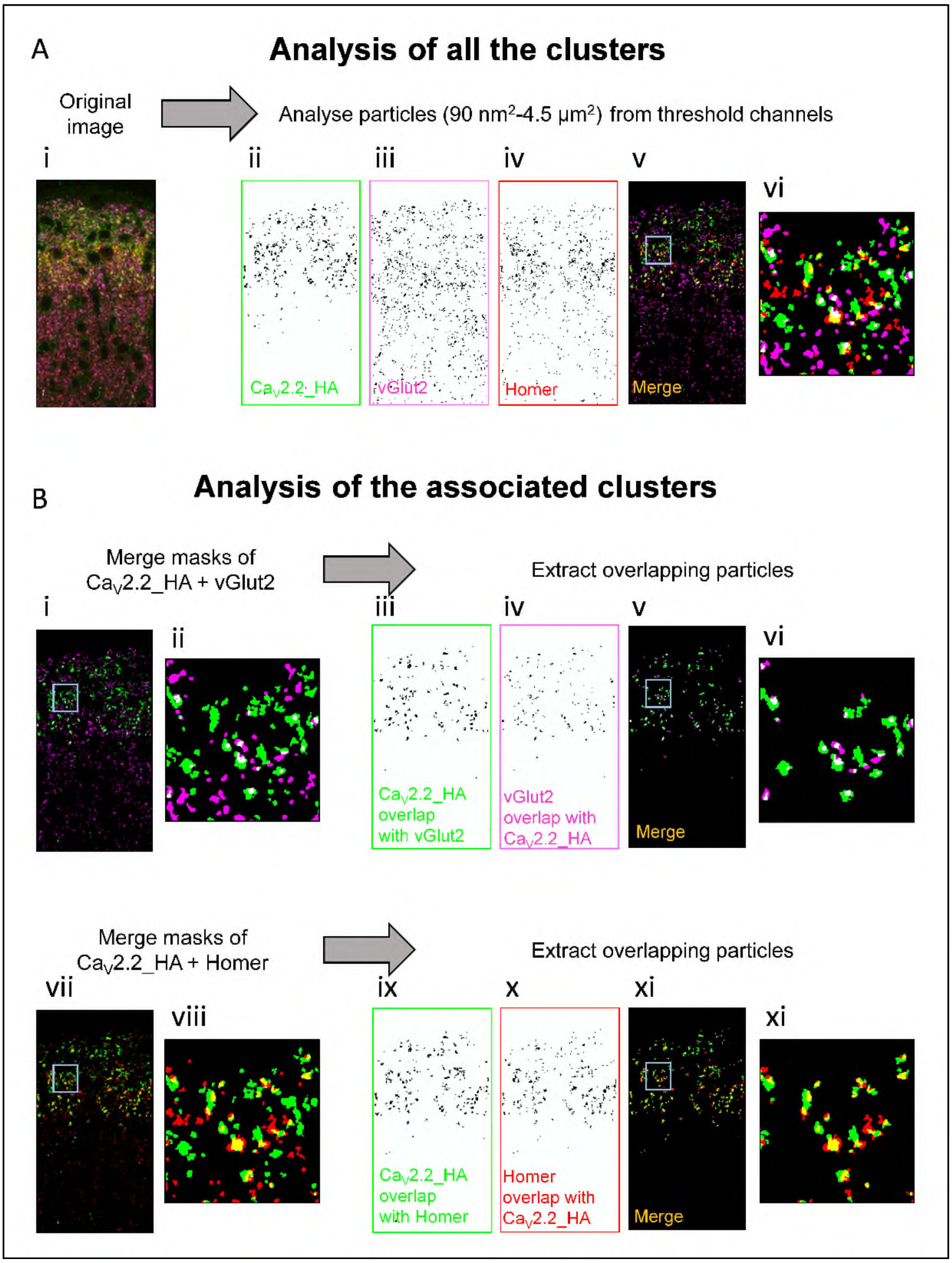

(**A, B**) Example of analysis of a super-resolution Airyscan image to select in first instance all the clusters positive for HA, vGlut2 and Homer (**A**) and the associated clusters (**B**). Starting from an ROI of 75 x 150 µm (Ai) labelled for HA (Green), Homer (Red) and vGlut2 (Magenta), each of the 3 channels was thresholded (auto-triangle threshold) and all the particles between 90 nm^2 and 4.5 μm^2 selected (Aii-vi). The merged masks of HA with vGlut2 particles (Bi, with zoomed region Bii) or HA with Homer particles (Bvii, with zoomed region Bviii) were used to extract the clusters that showed >1% overlap (Biii-vi and Bix-xi). Finally all particles were saved as list of ROIs to record their size and mean intensity from each corresponding channel of the original image (Ai).

Fig. S7 Box and whisker plots for Fig 4 I-M.

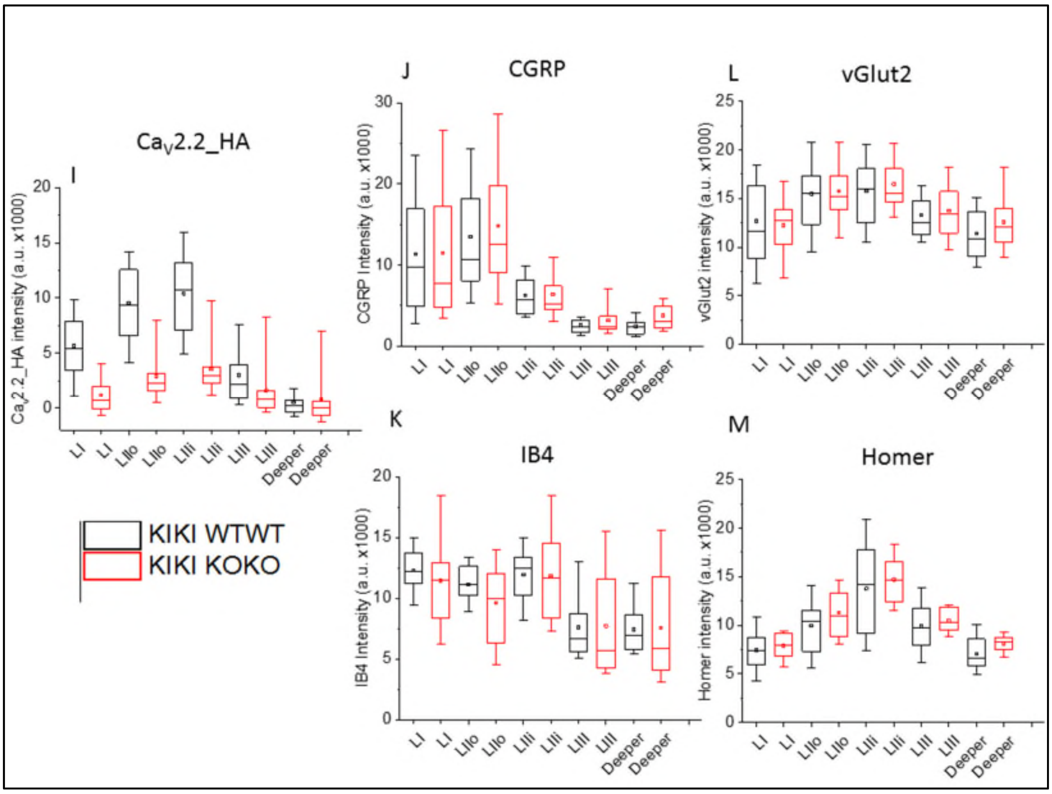

Same legend applies as for Fig. 4 I-M, except the plots represent box (25-75 %) and whiskers (10-90 %), with median represented as a line and mean represented as black or red square.

Fig. S8 Box and whisker plots for Fig 6 D-G

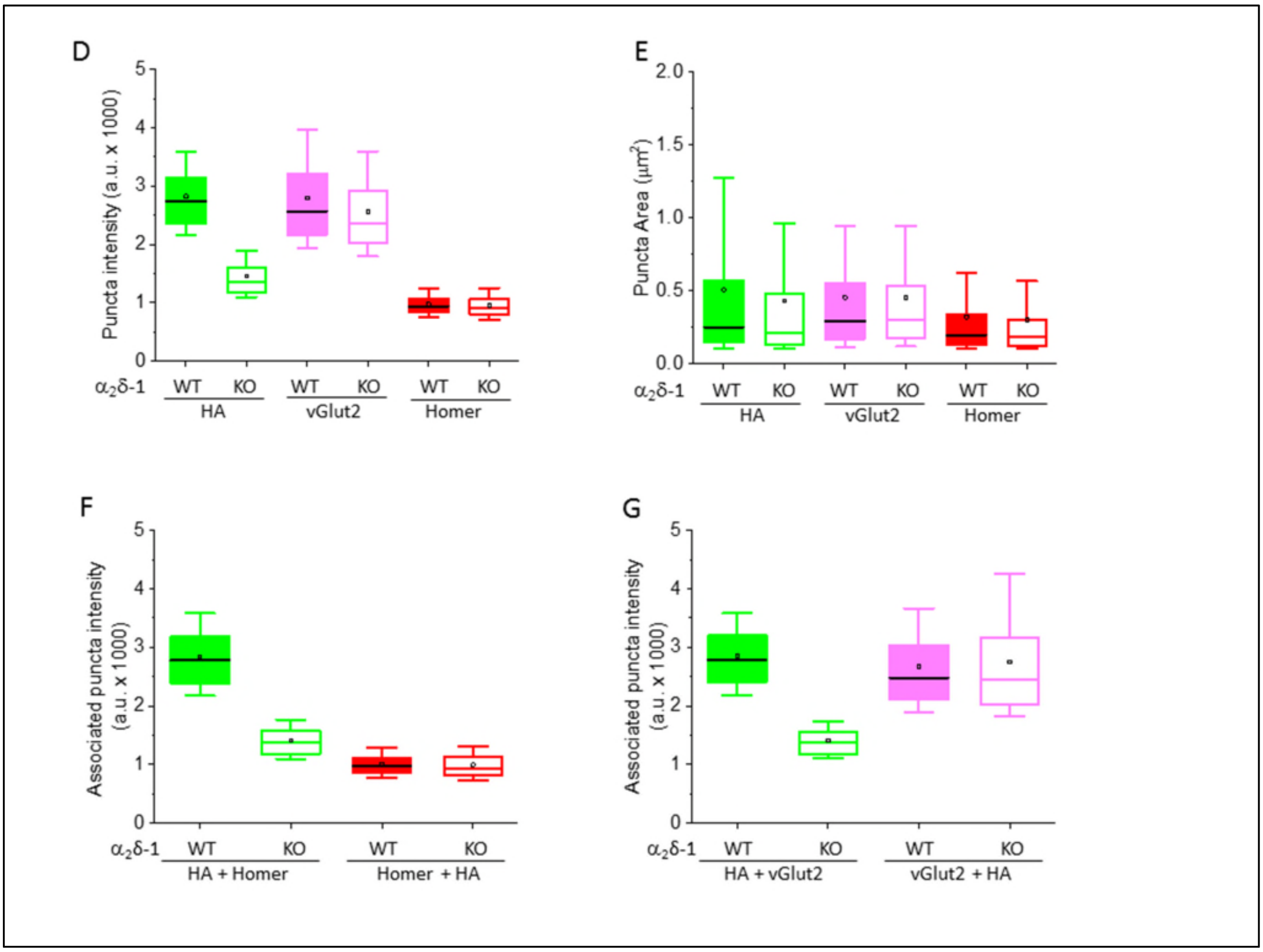

Same legend applies as for Fig. 6 D-G, except the plots represent box (25-75 %) and whiskers (10-90 %), with median represented as a line and mean represented as a black square.

Table S1 Body weights of mice of different genotypes

**Genotype**	**male body weight (g), (n)**	**female body weight (g), (n)**
$Ca_v2.2\_HA^{WT/WT}$	28.4 ± 0.7 (19)	21.2 ± 0.5 (16)
$Ca_v2.2\_HA^{KI/KI}$	27.2 ± 0.5 (22)	22.6 ± 0.6 (15)
$Ca_v2.2\_HA^{KI/KI}$; $\alpha_2\delta$-$1^{WT/WT}$	27.7 ± 0.8 (14)	20.0 ± 1.0 (6)
$Ca_v2.2\_HA^{KI/KI}$; $\alpha_2\delta$-$1^{KO/KO}$	23.6 ± 0.6 (9)	18.7 ± 0.4 (7)

The body weights of mice (9 - 11 weeks old), measured prior to experiments in this study

Table S2

Perfusion 1			Perfusion 2						Perfusion 3		
**Mouse**	**Experiment**	**ROIs**	**Mouse**	**Experiment**	**ROIs**	**Mouse**	**Experimen**	**ROIs**	**Mouse**	**Experiment**	**ROIs**
Ca_V2.2_HA$^{KI/KI}$ $\alpha_2\delta$-1$^{WT/WT}$ N1	HA+CGRP+ vGlut2	6xHA	Ca_V2.2_HA$^{KI/KI}$ $\alpha_2\delta$-1$^{WT/WT}$ N2	HA+CGRP	6xHA	Ca_V2.2_HA$^{KI/KI}$ $\alpha_2\delta$-1$^{WT/WT}$ N3	HA+CGRP	6xHA	Ca_V2.2_HA$^{KI/KI}$ $\alpha_2\delta$-1$^{WT/WT}$ N4	HA+CGRP+ vGlut2	6xHA
		6xCGRP			6xCGRP			6xCGRP			6xCGRP
		6xvGlut2									6xvGlut2
	HA+IB4+ vGlut2	6xHA		HA+IB4	6xHA		HA+IB4	6xHA		HA+IB4+ vGlut2	6xHA
		6xIB4			6xIB4			6xIB4			6xIB4
		6xvGlut2									6xvGlut2
	HA+Homer+ vGlut2	6xHA		HA+Homer	6xHA		HA+Homer	6xHA		HA+Homer+ vGlut2	6xHA
		6xHomer			6xHomer			6xHomer			6xHomer
		6xvGlut2									6xvGlut2
Ca_V2.2_HA$^{KI/KI}$ $\alpha_2\delta$-1$^{KO/KO}$ N1	HA+CGRP+ vGlut2	6xHA	Ca_V2.2_HA$^{KI/KI}$ $\alpha_2\delta$-1$^{KO/KO}$ N2	HA+CGRP	6xHA				Ca_V2.2_HA$^{KI/KI}$ $\alpha_2\delta$-1$^{KO/KO}$ N3	HA+CGRP+ vGlut2	6xHA
		6xCGRP			6xCGRP						6xCGRP
		6xvGlut2									6xvGlut2
	HA+IB4+ vGlut2	6xHA		HA+IB4	6xHA					HA+IB4+ vGlut2	6xHA
		6xIB4			6xIB4						6xIB4
		6xvGlut2									6xvGlut2
	HA+Homer+ vGlut2	6xHA		HA+Homer	6xHA					HA+Homer+ vGlut2	6xHA
		6xHomer			6xHomer						6xHomer
		6xvGlut2									6xvGlut2
Wild-type N1	HA+CGRP+ vGlut2	6xHA							Wild-type N2	HA+CGRP+ vGlut2	6xHA
		6xCGRP									6xCGRP
		6xvGlut2									6xvGlut2
	HA+IB4+ vGlut2	6xHA								HA+IB4+ vGlut2	6xHA
		6xIB4									6xIB4
		6xvGlut2									6xvGlut2
	HA+Homer+ vGlut2	6xHA								HA+Homer+ vGlut2	6xHA
		6xHomer									6xHomer
		6xvGlut2									6xvGlut2

Legend to Table S2: Summary of all spinal cord immunohistochemistry experiments.

A total of 4 $Ca_V2.2\_HA^{KI/KI}\alpha_2\delta\text{-}1^{WT/WT}$, 3 $Ca_V2.2\_HA^{KI/KI}\alpha_2\delta\text{-}1^{KO/KO}$ and 2 wild-type mice, from 3 different sets of perfusions were used to study the distribution of $Ca_V2.2\_HA$ in the spinal cord together with the pre and postsynaptic markers stated. From each perfusion, we performed 3 independent experiments, staining sections for HA with CGRP, IB4 or Homer (plus vGlut2 in all experiments from Perfusions 1 and 3), therefore 9 independent experiments in total for HA. In each experiment sections of $Ca_V2.2\_HA^{KI/KI}\alpha_2\delta\text{-}1^{WT/WT}$ with no primary antibodies, and wildtype sections in experiments from perfusion 2, were include as negative controls. For quantification, in each experiment 6 ROIs per sample (2 per section, 3 sections) were analyzed and pooled together according to staining and genotype. For each perfusion set, 10 weeks old $Ca_V2.2\_HA^{KI/KI}$ $\alpha_2\delta\text{-}1^{KO/KO}$ and $Ca_V2.2\_HA^{KI/KI}$ $\alpha_2\delta\text{-}1^{WT/WT}$ siblings were used in parallel for each of the 3 immunohistochemistry experiments. $Ca_V2.2\_HA^{KI/KI}$ $\alpha_2\delta\text{-}1^{WT/WT}$ N1, N2, and N4 were male and N3 was female. $Ca_V2.2\_HA^{KI/KI}$ $\alpha_2\delta\text{-}1^{KO/KO}$ N1 and N2 were male and N3 was female. Both control wild-type mice were female
